# Mapping moral injury: Development and feasibility of the Clinical Interview for Moral Injury—military version (CIMI-M)

**DOI:** 10.1371/journal.pone.0359553

**Published:** 2026-09-30

**Authors:** Mariëlle L. de Goede, Paul A. Boelen, Brett T. Litz, F. Jackie June ter Heide

**Affiliations:** 1 ARQ Centrum‘45, Diemen/Oegstgeest, The Netherlands; 2 Department of Clinical Psychology, Utrecht University, Utrecht, The Netherlands; 3 VA Boston Healthcare System, Boston, Massachusetts, United States of America; 4 Boston University and Boston University School of Medicine, Boston, Massachusetts, United States of America; University of Foggia: Universita degli Studi di Foggia, ITALY

## Abstract

**Objective:**

There is currently no clinical interview to assess exposure to potentially morally injurious experiences (PMIEs) and moral injury, the potential clinical outcome associated with PMIEs. To enable a person-centered assessment of PMIEs and moral injury in military veterans, we developed a structured, non-diagnostic clinical interview to support descriptive assessment, case conceptualization, and treatment planning, and conducted an initial evaluation of its feasibility, acceptability, and clinical utility.

**Method:**

The Clinical Interview for Moral Injury—Military Version (CIMI-M) was developed through an iterative, stakeholder-informed process. Questions from existing moral injury instruments were selected, adapted and supplemented to create an interview with a descriptive, theory-driven approach. Clinicians (N = 7) and military veterans (N = 8) at two Dutch trauma centers who conducted the CIMI-M were interviewed and completed questionnaires assessing the CIMI-M’s feasibility, acceptability, and clinical utility. Interviews were analyzed using the General Inductive Approach for qualitative evaluation data.

**Results:**

Mean evaluation scores were 7.63 for veterans and 8.41 for clinicians (10-point scale). Qualitative analysis of interview transcripts revealed that the CIMI-M helped veterans share their experiences, while clinicians found it valuable for fostering trust and planning treatment. Both groups agreed that the CIMI-M generated insightful content about PMIEs and moral injury. Based on feedback, several interview questions were revised or added.

**Conclusions:**

We found preliminary evidence that the CIMI-M is a feasible, acceptable, and clinically useful descriptive interview. It offers a potentially valuable treatment-planning tool that complements paper-and-pencil approaches that assess PMIEs and moral injury. Further evaluation, including in non-veteran populations, is needed.

## Introduction

During military missions, service members may be exposed to or involved in high stakes events that violate deeply held moral beliefs, also known as potentially morally injurious experiences (PMIEs) [1]. The concept of moral injury delineates the potentially impairing impact from such events. It is commonly defined as “the lasting psychological, biological, spiritual, behavioral, and social impact of perpetrating, failing to prevent, or bearing witness to acts that transgress deeply held moral beliefs and expectations” [1]. In recent years, a growing body of research has shown that deployed service members face an elevated risk of PMIE exposure, moral injury, and associated mental health problems [2]. Consequently, moral injury has gained recognition as a pressing clinical problem, leading to the development of various interventions [3].

Despite its clinical relevance, moral injury is still an emerging construct conceptually and empirically. One development is the now broadly recognized distinction between PMIEs as a precipitating event and moral injury as a psychosocial outcome, which were originally conflated [4]. Another development is the delineation by an international consortium of different domains of impact which together define the syndrome of moral injury [5]. These domains entail: (a) alterations in self- and other-perception, defined as disruptions in how individuals define themselves or the world with respect to what they or others are capable of; (b) alterations in moral thinking, which entails judging oneself or others harshly, moralistically, and with condemnation; (c) social impacts, defined as alterations in one’s degree of comfort with others, connectedness, and social acceptance or belonging, as well as changes in the frequency and quality of engagement with others; (d) moral emotions and moods, defined as predominant, pressing, and easily triggered moral emotions; (e) self-harming/sabotaging behaviors, defined as deliberate and nondeliberate behaviors that negatively impact functioning and impair health, personal safety, and quality of life or well-being; and (f) changes in beliefs about life meaning and purpose, defined as emptiness, purposelessness, and alterations in religious or spiritual beliefs or behaviors. Debate remains, however, around what does and does not constitute a PMIE [6], and whether and/or how moral injury is distinct from depression and posttraumatic stress disorder (PTSD) [7–9]. There is also ongoing debate about whether clinical assessment should be symptom-focused or functional [10], and about the boundaries between moral frustration, distress, and functionally impairing moral injury [9,11]. These unresolved issues have direct implications for how moral injury is assessed clinically, and underscore the need for flexible, descriptive assessment tools that do not presuppose diagnostic boundaries.

Several questionnaires have been developed to assess exposure to PMIEs and moral injury as an outcome. These include the Moral Injury Events Scale (MIES) [12], Moral Injury Questionnaire—Military Version (MIQ-M) [13], Expressions of Moral Injury Scale—Military Version (EMIS-M) [14], Moral Injury Symptom Scale—Military Version (MISS-M) [15], and, more recently, the Moral Injury Outcome Scale (MIOS) [5] and Moral Injury and Distress Scale (MIDS) [16]; for a critical review, see [17]. However, questionnaire measures have significant limitations. Firstly, they may not provide the depth and detail required to fully assess the parameters of exposure to PMIEs and various domains of impact. Current moral injury questionnaires, for instance, do not inquire about what specific moral beliefs or values were violated (such as care, fairness, or loyalty), which is relevant for treatment. Secondly, self-report questionnaires are subject to response biases (e.g., social desirability bias, over-reporting and under-reporting) and variable interpretations of items [18]. Thirdly, questionnaires require gist judgments of past behavior, the precision of which can be arguably enhanced by a give-and-take dialogue with a clinician. Lastly, they do not enquire after patients’ perceptions of the PMIE and its consequences or preferences for treatment.

A comprehensive structured clinical interview facilitates a more complete understanding of an individual’s difficulties and helps identify concerns that patients may struggle to articulate. It uses prompts and follow-up questions to clarify details and ambiguous answers. A clinical interview is also a potentially more reliable way to assess the clinical significance of a presenting problem, making it an indispensable tool in treatment planning. Within the field of psychotrauma, most clinical interviews (such as the Clinician-Administered PTSD Scale for DSM-5; CAPS-5) [19] are designed to assess the presence and severity of symptoms in relation to diagnostic criteria. While such interviews are well suited for diagnosing established disorders, they are less fitting for assessing experiences and impacts that fall outside of symptom-based classification systems, such as moral injury. Because there is currently no consensus regarding the boundary conditions of moral injury, a diagnostic clinical interview is thus neither feasible nor desirable at this stage of construct development. Instead, a clinical interview that adopts a descriptive approach to the assessment of PMIEs and moral injury is more appropriate. A descriptive clinical interview allows for a systematic exploration of the individual’s lived experience, including the meaning attributed to PMIEs, violated moral values, and resulting emotional, relational, and existential impacts—domains that may not be structurally assessed in a regular intake interview. An example of a descriptive clinical interview not rooted in diagnostic criteria is the Cultural Formulation Interview (CFI) [20], which was designed to assist clinicians in identifying the impact of culture on illness and ensure cultural sensitivity throughout the diagnostic process. Rather than assessing symptoms and generating diagnoses, it facilitates a deeper clinical understanding of the presenting problem by clarifying the patient’s perspective, relevant contextual factors, and expectations for care, thereby supporting informed treatment planning. As such, it presents a valuable example for the assessment of moral injury in the absence of established diagnostic criteria.

Despite the rapid development of therapeutic interventions and self-report instruments for moral injury, currently no clinical interview for moral injury exists. To fill this gap, we developed the Clinical Interview for Moral Injury—Military Version (CIMI-M) as a descriptive clinical tool to assist case conceptualization and treatment planning. Our objective was to create a clinical interview for moral injury that: (1) maps exposure to different PMIE types as well as the impact of such experiences across the six moral injury domains identified by Litz et al. [5]; (2) allows for a descriptive dialogue that includes the patient’s own perspective; and (3) facilitates case conceptualization, treatment planning, and an exploratory configural caseness determination. We also aimed to produce an interview that would be readily trainable, thereby providing a practical and broadly useable tool. In this paper, we describe the development of the CIMI-M. We also provide the results of a preliminary feasibility study of the CIMI-M, which entailed an exploratory examination of veterans’ and therapists’ perspectives of its initial feasibility, acceptability, and clinical utility, and which was used to modify the interview. We hypothesized that both veterans and clinicians would judge the CIMI-M as feasible, acceptable and clinically useful.

### Development of the CIMI-M

#### Setting.

The CIMI-M was developed at ARQ Centrum’45, a Dutch national center for expert diagnostics and treatment of complex psychotrauma. The center provides specialized care for military veterans as part of the Dutch Veterans Health Care. 59% of veterans at the center present with a moral injury or moral injury plus PTSD symptom profile [21].

### Instrument aim

The CIMI-M was designed as a tool for case conceptualization and treatment plan for moral injury. A case conceptualization entails integrating a patient’s individual experiences, and describing and making sense of a patient’s problems, within a treatment framework [22]. Conceptualization is a collaborative process between the therapist and patient that honors the individuality of the patient and results in a parsimonious overview of any information considered essential to guide treatment planning [23]. Beyond supporting case conceptualization and treatment planning, the CIMI-M could also be used to determine probable clinical caseness. We experimentally defined clinical caseness as: (1) endorsement of at least one high-stakes and currently distressing PMIE; (2) reports of functionally interfering problems in at least two domains of moral injury; (3) that are new onset; and (4) for which recovery has been impeded or is unlikely without clinical intervention. This configural decision rule for caseness is by definition not a formal diagnosis of a mental disease (moral injury is not currently in a disease taxonomy), but it formalizes the assessment of whether an individual warrants treatment for a potential moral injury within the context of data collected in the CIMI-M.

### Design

We planned the development process based on recommendations for intervention development by O’Cathain et al. [24], while also incorporating Haynes et al. [25] guidelines for content validity. Due to the absence of formal guidelines for developing a descriptive clinical interview, we adapted the process proposed by O’Cathain et al. [24] to develop the CIMI-M, as these principles for intervention development were well-suited to our objectives and provided a framework for a comprehensive and systematic development process. Steps taken included: (1) a literature review; (2) defining the construct and determining a theoretical framework; (3) initial selection of questions and prompts; (4) expert consultation; (5) piloting the interview; (6) conducting a feasibility study; and (7) refining the interview. We approached the process in a dynamic way, moving back and forth between overlapping actions through iterative cycles. We also involved various stakeholders (enrolled patients, patient representatives, therapists, and moral injury researchers) throughout the process. Development ran from June 2022 until January 2025.

### Literature check

To ascertain the absence of a clinical interview for moral injury and collect potential interview questions, we conducted a search on PubMed and APA PsycINFO for peer-reviewed papers, using the search terms “moral injury AND (interview OR clinical interview OR diagnostic interview OR clinician-administered OR clinician-rated OR intake OR qualitative)”. We discovered no extant clinical interviews for moral injury, with only scant references to interview questions used in qualitative studies (with questions directed at veterans or clinicians [5,26–28]. In addition, we reviewed several moral injury-related (work)books and manuals that included suggested questions for clinicians [29–32]. We also received interview questions from ongoing studies.

### Theoretical framework

To ground an intervention (or in this case, a clinical interview) in theory, O’Cathain [24] recommends drawing from more than one existing theory, as multiple theories can help identify what is important and feasible. Moreover, drawing from multiple theories can avoid confirmation bias among clinicians who may focus on answers supporting their favored treatment [33]. We therefore incorporated the following theories of moral injury into the interview: (1) the working model of moral injury by Litz et al. [1], which centers around attributions made about moral violations, (2) the functional approach by Farnsworth et al. [34], which focuses on the role of coping with moral pain, and (3) the six domains of moral injury delineated by Litz et al. [5] (namely, self-perception, moral thinking, social impacts, self-harming/self-sabotaging, impairing moral emotions, and beliefs about meaning and purpose). In the final interview, each theory informed distinct components of the interview: the working model of Litz et al. [1] underpins the questions about attributions made in relation to the PMIE; the functional approach of Farnsworth et al. [34] informed questions about coping with moral pain and self-harming or self-sabotaging behaviors; and the six-domain framework of Litz et al. [5] provided the organizing structure for the impact section of the interview.

In the instruction manual, we included information about the moral foundations theory [35], to help the interviewer examine what moral values were violated during a PMIE, and the definition of a PMIE by Bonson et al. [6]. This definition delineates a PMIE as a high-stakes event in which “one’s deeply held beliefs of right and wrong are at stake,” irrespective of the outcome. According to this framework, PMIEs may or may not meet the PTSD A-criterion [7] but invariably involve a violation of moral values and/or a conflict with moral beliefs.

### Initial item selection and generation

The literature review led to an initial pool of 201 open-ended questions, which the first and fourth author clustered according to a structure that was refined over several iterations. The initial structure included the following headings: general/opening questions, PMIE(s), general impact, moral emotions, cognitive processes (self-perception, moral thinking, beliefs about meaning and purpose), coping (general coping, self-harming/sabotaging), social impacts, functional impacts, desired change/readiness for change. Within this item pool, whenever two questions were nearly identical, the clearest and most concise question was retained. If two questions inquired about the same experience but were worded very differently, both were kept. Questions that were deemed missing were added. This resulted in a list of 116 items.

### Expert consultation and initial testing

Three patient representatives and three moral injury scientist-practitioners from the Netherlands and the United States were asked to provide feedback on the initial list of 116 questions, arranged according to the abovementioned structure, in an Excel file. They were instructed to mark questions as essential or redundant, to add any questions they deemed missing, to comment on the preliminary structure, and to provide any other feedback in a column behind each question or heading. Questions that they marked as essential were included in the first interview draft. Disagreements (i.e., one person marking a question as essential, another as redundant) were solved through discussion between the first and fourth author, after which the second and third author provided additional feedback. Some questions were retained as potential follow-up questions. This resulted in the first interview draft, consisting of 32 questions and 31 potential follow-up questions. Questions about the impact of a PMIE were linked to an index PMIE by adding “as a result of [the experience]” to the question (e.g., “Do you have a tendency to undermine or sabotage yourself as a result of [the experience]?”). An introduction with instructions for administration was also added.

The first draft was then evaluated by another five experts (clinicians and researchers with knowledge of moral injury and instrument development) and one patient representative. They were instructed to evaluate the introduction and the wording of the questions, to mark redundant questions, and to include any other feedback. Disagreements in the feedback were again solved through discussion between the first and fourth author. This led to a second draft, which was then tested with two veterans undergoing treatment at ARQ Centrum’45. Following this, the interview underwent further refinement, resulting in the version used for the feasibility study. This refinement included removing the phrase “in the past month” from questions regarding the impact of the PMIE, and changing those questions from the past to the present tense (e.g., “In the past month, did you have a tendency to undermine or sabotage yourself, as a result of [the experience]?” to “Do you have a tendency to undermine or sabotage yourself, as a result of [the experience]?”). Initially included to ensure that experienced problems were current, this phrasing was ultimately seen by both veterans and clinicians as making the interview feel more like a checklist than a dialogue, thus undermining one of the interview’s primary objectives.

The final version used for the study included 26 questions and 36 potential follow-up questions, grouped under the abovementioned headings, as well as an introduction and instructions.

## Methods

### Design

We then conducted a feasibility study using qualitative (interview) and quantitative (descriptive) data. The study concentrated on the perceived initial feasibility, acceptability, and clinical utility of the interview. Feasibility refers to the extent to which the interview can be practically administered in the intended clinical context; acceptability refers to the degree to which the interview is experienced as appropriate, reasonable, and tolerable by both veterans and clinicians; and clinical utility refers to the extent to which the interview generates information that supports clinical decision-making, case conceptualization, and treatment planning.

### Setting and participants

The study was carried out at two centers offering tertiary care to trauma-exposed military veterans: ARQ Centrum’45 and Sinai Centrum. Recruited were (1) veterans who were on the waitlist for treatment and who, during intake, had disclosed a PMIE and/or negative moral emotions related to a traumatic event, and (2) clinicians familiar with the concept of moral injury. When a veteran was already being seen by a clinician sufficiently familiar with moral injury, that clinician was invited to participate; where this was not the case, an alternative clinician with relevant expertise was approached. All clinicians who participated followed a general in-company training on moral injury. Additionally, prior to administration, all clinicians received the CIMI-M including the instructions manual and met individually with the first author to discuss the interview and the study procedure. The CIMI-M was conducted 10 times. Eight veterans consented to participate. Two veterans who did not want to participate in a debrief interview consented to their clinician being interviewed for the study. Seven clinicians took part in the study, two of which conducted the CIMI-M twice. One clinician, as the study coordinator, conducted the CIMI-M too but was not interviewed. The final sample thus consisted of 15 participants, which is deemed sufficient for feasibility testing [24]. The veterans consisted of seven men and one woman; the clinicians consisted of six women and one man. All participants had the Dutch nationality. Of the veteran participants, three had a multicultural background.

### Procedure

The study received ethical approval from the Ethics Review Board of the Faculty of Social and Behavioral Sciences of Utrecht University [approval no. 23–0026]. The recruitment period for the study ran from 17/07/2023 until 14/09/2023. Veterans who were potential participants were contacted by either the first author or by their primary therapist; clinicians who were potential participants were contacted by the first author. In both cases, the study procedure was explained and an information letter shared. The information letter stated that any personal and/or clinical information shared during the CIMI-M was confidential and would only be used for clinical purposes. If veterans and/or clinicians agreed to conduct the CIMI-M, clinicians were asked to schedule an appointment with the veteran for conducting the clinical interview in consultation with the first author, who then scheduled appointments to conduct the debrief interviews with the veteran and clinician on the same day. Clinicians were instructed to schedule 90 minutes for the CIMI-M, based on prior testing. Some clinicians already knew the veteran they interviewed, while others did not. After conducting the CIMI-M, veterans and clinicians separately met with the first author and signed two informed consent forms (one for the study and one for recording the debrief interview). Subsequently, they filled out the debrief questionnaire and took part in the semi-structured debrief interview.

Veterans’ answers to the CIMI-M were used for treatment planning and some subsequently received treatment for moral injury. All clinicians conducted a follow-up session one to two weeks later to discuss the case conceptualization and its implications for treatment with the veteran.

### Assessment

Veterans completed the Debriefing Instrument for Patients (DIP), and clinicians completed the Debriefing Instrument for Clinicians (DIC) [36]. The DIP and DIC consist of 14 and 17 self-report items respectively, assessing the clinical utility (DIP 8 items, DIC 11 items), feasibility (3 items), and acceptability (3 items) of a clinical interview. Items are rated on a 4-point Likert-scale (*strongly disagree, disagree, agree, strongly agree)* plus the option of *not applicable*. The questionnaires were originally developed to evaluate the CFI [36]. The questionnaires were translated to Dutch and some of the wording was adapted for study purposes, as approved by the authors. We removed two negatively worded DIP items (10 and 12) as prior research demonstrated they negatively impacted internal consistency [36], resulting in two items for feasibility and two for acceptability. Additionally, during the debrief interview veterans and clinicians were asked to rate the CIMI-M overall on a 10-point scale ranging from 1 (*very low*) to 10 (*very high*) (“How would you evaluate the interview on a scale from 1 to 10 and could you explain this score?”).

Semi-structured debrief interviews were conducted to obtain more detailed information about how veterans and clinicians experienced the CIMI-M. Questions were partially based on interview questions used for the evaluation of the CFI [36]. Included were questions assessing the most and least helpful questions of the CIMI-M, its structure and length, its role in treatment planning, overall interview quality, and suggestions for improving the interview. Debrief interview questions are included as a supplement in S1 File Supporting Information. Debrief interviews lasted 20–30 minutes.

### Analysis

Descriptive statistics were computed using SPSS version 23 for Windows. DIP/DIC responses were used to generate descriptive data. There were no missing DIP/DIC data, and one missing response for the overall CIMI-M rating. Answers to the debrief interview were inserted in MAXQDA 2022 and analyzed by the first author using the General Inductive Approach for coding qualitative evaluation data [37]. Analysis proceeded in four steps. First, all text segments relevant to the evaluation objectives were identified and marked. Second, relevant segments were labeled to create an initial set of categories, producing 27 codes. Third, codes were reviewed and merged to minimize redundancy and overlap, resulting in 10 labels. For example, the codes ‘reflection on experiences’, ‘awareness of the impact’, and ‘new insights’ were merged into the label ‘reflection and insight’. Fourth, we grouped these labels into overarching categories reflecting the evaluation objectives of feasibility, acceptability, and clinical utility. The evaluation objectives guided the focus of the analysis without imposing a priori expectations about specific findings, consistent with the General Inductive Approach [37].

## Results

### Descriptive statistics

Veterans rated the CIMI-M favorably, with an average rating of M = 7.63 (SD = 1.16, range 5–9). On the DIP, clinical utility was rated M = 3.34 (SD = 0.47, range 2–4), feasibility M = 3.39 (SD = 0.55, range 2–4), and acceptability M = 3.33 (SD = 0.75, range 2–4).

Clinicians rated the CIMI-M very positively, with an average rating of M = 8.41 (SD = 0.38, range 7.50–8.50). On the DIC, clinical utility was rated M = 3.61 (SD = 0.22, range 2–4), feasibility M = 3.38 (SD = 0.41, range 3–4), and acceptability M = 3.29 (SD = 0.49, range 2–4).

### Qualitative analysis

Through qualitative analysis we identified several themes related to various aspects of the interview. We divided these themes into several categories related to the interview’s feasibility, acceptability, and clinical utility. Sample quotations per theme are listed in Table 1.

**Table 1:** Sample quotations per category and theme.

Category & theme	Sample quotation
Feasibility: *Training*	Clinician: “I lean towards saying that it is advisable to have some information about moral injury. So in that sense—just like you have a training for the CAPS—I would also like to have a training for this. It doesn’t have to be that long at all, but it is important that someone understands when we are truly speaking of a morally injurious experience and when we are not.”
*Interview structure*	Veteran: “For an interview it is quite long, yes […] It’s not bad, it’s necessary, to cover all the questions properly and also certainly given the emotions… then you just need time, you can’t do that in a ten-minute interview.” Clinician: “The structure is logical, you start somewhat broader and then zoom in more and more on, well, the things of which someone says, ‘that is morally distressing me’. So I think it’s nice for the person you’re interviewing to work with such a ‘funnel’. And that you don’t go straight to the most painful part, but first explain something and start broader, and then look at ‘what is bothering me the most and how does that manifest.’”
*Interview manual*	Clinician: “I do think it’s an easy interview to conduct, also with the instructions in the manual. Sometimes you have semi-structured interview manuals, where you have to look in three places at once in order to follow the interview properly or to not skip things. In that respect I just found this very practical.”
Acceptability: *Emotional impact*	Veteran: “This touched me in such a way that I see the images again, I was back again in that moment, and I could also interpret the moment and distance myself from it, so in the sense of, ‘okay, that was then.’ Still what I feel started in that moment and isn’t pleasant. And it [the interview] evoking all these things in me wasn’t pleasant either. […] It was heavy in the sense that you recall many things that you have been avoiding, for me almost for 30 years. I had all the space I needed to tell that story. And also… I also felt acknowledgement… ‘it’s not crazy that I am bothered by that’. That feeling came over me strongly.”
*Interview questions*	Veteran: “It also gave me a calm feeling to think about what you don’t always think about. The film [of the PMIE] often plays in my mind, but then you don’t think about what is happening now or how you feel. And that, because of those questions you asked, I did get the idea of, ‘yeah, how do I actually feel now?’” Clinician: “Because of those questions, the probing like, ‘what happened, what happened then, what did you do, what did others do’ and so on—if you ask all of that, people can no longer avoid [what happened]. For example, the man I spoke to, of course he was quite ashamed of what he had done, so he didn’t say it at first, but in the end he did share that he had killed someone. So I found that quite useful.”
*Therapeutic relationship*	Clinician: “I do think it [the interview] immediately did something with the trust. I had the feeling that, well… for me it was also our first introduction—I am going to be his primary therapist—but we were able to go into quite a bit of depth right away. And I was also able to feel how much he already was willing to give me so to speak, how much he confided in me, how open he was already.”
Clinical utility: *Sharing their story*	Veteran: “I liked this better than the CAPS, because I actually found it to be more aligned or smoother, [...] which made it feel more like a conversation than like ticking items off a questionnaire. Because that feeling is… I generally experience that as very unpleasant. Because that gives me the feeling that you’re a number, instead of… being able to tell your story and that it matters.” Clinician: “I think we went into depth in a structured way without anyone feeling like we’re ticking things off a list. Which of course is the case with the CAPS, but this [interview] is also like, ‘I want to understand better how this has shaped you and how you this still bothers you’. So I think that for people who primarily experience a lot of distrust, it is a really beautiful way to say, ‘hey, I want to understand you and I want to take a good look at from which angle you can best be treated.”
*Reflection and insight*	Veteran: “The questions are actually the right ones. That’s really nice on the one hand, but not on the other, because you stir up a lot more than I actually thought was there. So you also get to know yourself a little bit more; you actually also learn how far along you are.” Clinician: “I’ve got the idea that we really gained more insight into his symptoms and also the role of the morally traumatic event. Because he talked about it quite rationally, especially at first, and the interview made his feelings come to the surface, as well as more awareness of how it influences his daily life. So insight into his feelings, but also his cognitions and that self-care for him, ‘that it really caused me to take less good care of myself and to work against myself’. And also the realization that other people failed to do something, he found that important too, as in, ‘that is something I’m still quite angry about actually.” And that anger is still a theme in his life, so that all gave him a lot of insight.”
*Concept of moral injury*	Clinician: “I liked the fact that at the beginning [of the interview] there is an explanation for yourself to clearly distinguish between the PMIE and the moral injury, the impact. Because in practice, now that the concept is receiving more clinical attention, I still see that the event itself is equated with the outcome, that is it equated with moral injury.”
*Relation to treatment*	Clinician: “I think, for a number of my patients, if I had done this interview before the [online treatment] module, the module would also have run more smoothly. Because I notice now that the module can now take a very long time—which is not necessarily a bad thing because it does support treatment—but that’s not necessarily what the module is for. And so suppose I had conducted this first, then maybe we would have gotten through the first chapters [of the module] faster. [...] Because now I spend quite a lot of time going over the explanations, because I don’t yet know at all what their moral values are. Whereas if you have already conducted this interview, then you can reflect on that and you can actually zoom in directly on: ‘which morally injurious incidents are the most painful for you?’”

### Feasibility

#### Training.

Some clinicians recommended developing a brief training for interviewers, to ensure that interviewers would understand the concept and properly use the interview, while also familiarizing them with its structure.

### Interview structure

The interview structure, including its sections and the order of questions, was generally seen as logical and appropriate. Length and duration (approximately 1.5 hours) were almost unanimously deemed sufficient. While some veterans found the interview lengthy, they acknowledged that a shorter interview would not have allowed them to share their stories adequately, particularly when it might be a veteran’s first time (fully) sharing the story of the PMIE.

### Interview manual

Overall, clinicians were positive about the interview manual, including its instructions, lay-out and readability. Some suggestions for improvement were given, including the addition of a template for the case conceptualization.

### Acceptability

#### (Emotional) impact.

Veterans reported experiencing difficult emotions and physical reactions, such as heavy breathing and reliving their morally injurious experiences in response to the interview. While many found the interview heavy and challenging, most indicated that confronting these emotions felt necessary. Some appreciated the clinician’s empathetic response as a recognition of their moral pain. Clinicians confirmed observing these reactions in the veterans.

### Interview questions

Veterans found the interview questions easy to understand and free of judgment. The question about the emotional impact of the PMIE was generally found most helpful: despite evoking difficult emotions, some said it helped them articulate their feelings. Conversely, many veterans found a question about the spiritual impact of the PMIE less helpful, as they could not relate to it. Veterans found the interview contained the right level of detail. One veteran struggled to focus on one incident, as he had experienced multiple PMIEs.

Clinicians offered suggestions for refining questions and reducing potential overlap. Clinicians saw the questions on military context as particularly helpful. They also particularly appreciated the section on moral emotions and cognitions. Clinicians also deemed the question about the spiritual impact of the PMIE the least helpful, as well as a question about the veteran’s quality of life, which was considered redundant.

### Therapeutic relationship

Veterans stressed the importance of a clinician fostering trust and safety. Clinicians maintained that the interview enhanced the therapeutic relationship and built trust, making it a valuable precursor to moral injury treatment if conducted by the same clinician. Some clinicians believed another therapist could also administer the interview.

### Clinical utility

#### Sharing their story.

Veterans mentioned that sharing their story was hard but found that the interview helped them express their experiences and the subsequent impact, both during the study and subsequent treatment. Clinicians similarly believed the interview helped veterans to open up, as the interview included in-depth questions and inquired about the meaning attributed to the PMIE. Several clinicians and veterans appreciated that the CIMI-M felt more like a natural conversation than a checklist.

### Reflection and insight

Veterans indicated that the interview helped them reflect on what happened to them and brought new insights. Some mentioned typically avoiding thinking or talking about the PMIE but found that the interview helped them view the event in a new light (e.g., realizing “it’s not strange that this has had such a profound impact on me.”). Clinicians reported gaining deeper insight into the meaning attributed to the PMIE, its impact on daily functioning, and what moral beliefs were violated.

### Concept of moral injury

Some clinicians expressed difficulty in fully grasping the concept of moral injury and how to define a PMIE. They said the interview helped them to better understand the concept and what questions to ask.

### Relation to treatment

Veterans were hopeful that sharing their story would help them get the right treatment. Clinicians remarked on the timing of the interview, seeing value in conducting the interview during the intake phase or immediately before the start of trauma treatment with veterans who have experienced a PMIE. Some clinicians expressed having a better idea of what treatment to offer following the interviews; others remained uncertain and asked for intervention suggestions at the end of the interview.

### Interview adaptation

Based on the feasibility study, we made several refinements to the CIMI-M to produce its final version. Revisions included the rewording of some questions, additional follow-up questions, and a new question about self-worth. For example, a question about emotional coping strategies was initially followed by only one follow-up question probing whether the coping behavior was helpful. Based on feedback, this was revised to include several more specific follow-up questions inquiring about coping behavior in different contexts, such as when alone or in interactions with others. Additionally, as a question about the spiritual impact of the PMIE did not resonate with non-religious veterans, we split this question into two questions: one inquiring about the impact of the PMIE on religious and spiritual beliefs and another inquiring about the loss of belief in the meaning or value of life. We also made structural adjustments to the headings and question sequencing for the interview to align with the domains of moral injury outlined by Litz et al. [5].

The resulting structure included the following headings: section 1: context; section 2: the PMIE(s); section 3: domains of moral injury (subheadings: impairing moral emotions, self-perception, moral thinking, self-harming and self-sabotaging, social impacts, beliefs about meaning and purpose, and impact on other areas of life); section 4: desired change and readiness for change. Section 4 assesses the veteran’s readiness for treatment and their preferences for care (e.g., preference for individual versus group therapy or including chaplain care), to support a person-centered approach to treatment planning. Moreover, because treatment for moral injury can be challenging (requiring veterans to confront deeply painful experiences they may have been avoiding for years), understanding a veteran’s readiness for change at the outset provides clinically valuable information [32]. We also revised the introductory text and appended a case conceptualization template at the end of the interview, based on the interview structure. The final CIMI-M therefore consisted of: 1) an introductory section for clinicians including guidance on administering the interview; 2) an introductory text to be read aloud to the veteran at the start of the interview; 3) the interview itself, organized according to the headings outlined above; and 4) a template to support case conceptualization and writing a clinical report. The full CIMI-M is provided as a supplement in S2 File Supporting Information.

## Discussion

We reported on the development and preliminary evaluation of the Clinical Interview for Moral Injury (CIMI-M), a structured, descriptive, and easily trainable interview designed for use with (former) service members to support case conceptualization of their moral injury. The interview was developed through an iterative process that involved moral injury experts (i.e., patient representatives, clinicians, and researchers) and was evaluated in a feasibility study. The selective sample of clinicians and veterans who participated in this preliminary evaluation deemed the CIMI-M feasible, acceptable, and clinically useful. Its descriptive approach aids in mapping the impact of PMIE exposure, context of the PMIE, violated moral values, and meaning attributed to the event. In this way, the interview clarifies the patient’s perspective and complements existing self-report scales for moral injury, thereby supporting more informed treatment planning and intervention selection.

The development of the CIMI-M presented a challenge, given the ongoing debate around the construct of moral injury and its clinical boundaries. The lack of conceptual clarity extends to core aspects of moral injury, such as the definition of PMIEs [6] and the distinction between clinically significant moral injury and normative distress [11]. Consequently, the interview does not offer a cut-off point for determining clinically significant moral injury nor does it produce a severity score. As a result, it cannot be used to “diagnose” moral injury or to track changes in morally injurious impacts, unlike clinical interviews such as the CAPS-5. However, we included exploratory instructions for caseness determination in the interview manual, to provide clinicians with a structured basis for deciding whether an individual’s difficulties are of sufficient severity and specificity to warrant treatment for moral injury. Future research should examine the interrater reliability of this decision rule, and its clinical validity; for instance, whether caseness determinations made using the CIMI-M accurately identify individuals who benefit from moral injury treatment, and whether they show adequate agreement across clinicians.

The CIMI-M addresses the lack of a consensus definition by design, by integrating multiple theoretical frameworks, making the CIMI-M a theoretically inclusive instrument that addresses the diverse and evolving conceptualizations of moral injury. Simultaneously, the current development of the CIMI-M helps clinicians to address moral injury in real time, rather than waiting for a conceptual consensus to emerge. Many service members and veterans require immediate support for moral injury, creating a need for practical tools that facilitate care in the absence of a fully agreed upon conceptualization. Additionally, feedback from veterans suggested that a diagnostic approach would be less effective in creating the supportive environment needed for sharing their story, especially if for the first time. Moreover, for clinicians wishing to identify *clinically significant* moral injury, existing self-report instruments may be employed alongside the CIMI-M, such as the MIDS, for which cut-off scores have been estimated [11]. By clearly identifying the presence of a PMIE, assessing moral values that were violated, and determining to what extent current problems across moral injury domains are attributable to these PMIEs also helps distinguish these problems from PTSD and depression. It must again be noted, however, that creating a diagnostic tool was not the aim of the CIMI-M, as moral injury is not a classified mental disorder. Given these factors, we believe that the development of the CIMI-M was timely and important, providing a meaningful framework for addressing moral injury even as the field continues to refine the construct.

Although this study showed promising preliminary results, the CIMI-M should be regarded as a prototype at this stage of development. The CIMI-M is not a continuous symptom severity (or frequency) scale, consequently, conventional psychometric indices such as internal consistency reliability and construct validity are not germane. However, future research should examine interrater reliability, cross-cultural content validity, and criterion-related validity with respect to concurrent and predictive validity. For example, trained raters could independently review the same recorded CIMI-M interviews and complete the reporting template. Agreement could be evaluated for categorical determinations such as PMIE presence/absence, PMIE type, and presence/absence of clinically salient impact within each domain using a weighted kappa to determine agreement relative to chance. With respect to concurrent validity, the CIMI-M could be administered to various groups and the caseness variable could be compared with continuous scores on a moral injury outcome scale. An odds ratio per unit increase in scale score could index the strength of association and the predicted probabilities of caseness across score ranges could be determined.

### Strengths and limitations

As the first clinical interview for moral injury, the CIMI-M fills a significant gap in the assessment of moral injury. The CIMI-M being theoretically inclusive, clinicians working from different theoretical perspectives or trained in different therapeutic modalities for moral injury are likely to benefit from using the CIMI-M. Using an iterative feedback process allowed for continual refinement of the CIMI-M based on expert and patient feedback, incorporating diverse insights to ensure the interview is patient-centered, user-friendly, and efficient. Engaging the perspective of those with experiential knowledge throughout the development process also helped ensure the interview is not only theoretically sound but also attuned to the needs of the target population [38]. The preliminary evaluation also included interviews with both veterans and clinicians, strengthening the reliability of the findings and contributing to a more nuanced understanding of the CIMI-M’s initial acceptability, feasibility and utility in clinical practice.

However, there are also several limitations to the process through which the CIMI-M was developed. Firstly, a limitation encountered during the development process is the absence of formal guidelines specifically tailored to the creation of descriptive clinical interviews. While the adaptation of O’Cathain et al.’s [24] intervention development framework provided a systematic approach, the lack of specialized guidance for developing a clinical interview might have led us to overlook aspects unique to clinical interviews. Secondly, interview questions were generated primarily by adapting items from existing interviews and seeking expert feedback on these adaptations. This approach has its limitations, as it relies on existing literature and expert opinion, both of which can vary with regards to the conceptualization of moral injury [5]. Thirdly, the iterative feedback process through which the CIMI-M was developed, while thorough, involved a relatively specialized group of stakeholders who primarily concentrated on ensuring the questions were appropriate for a military population. This limits the generalizability of the CIMI-M across different populations. Particularly the context-related questions, which inquire about the interviewee’s deployment(s) and reasons for joining the military, would require modification if the CIMI-M were to be adapted for civilians or individuals in other professions. Additionally, the feasibility study was conducted at two Dutch centers offering tertiary care to trauma-exposed military veterans, who in this study were mostly male. Therefore, to determine whether the acceptability, feasibility, and clinical utility of the CIMI-M can be generalized across various settings and populations, more systematic research is required, preferably across different countries, military cultures, and healthcare systems, with participants of different genders and backgrounds. An international field trial, similar to the one conducted for the CFI [36], would be particularly valuable in this regard. More firmly establishing the clinical utility of the CIMI-M would also necessitate long-term data collection, ideally extending post-therapy, to assess if and how the interview improves case formulation, treatment matching, or clinical outcomes.

Another limitation concerns the use of the DIP and DIC, which were developed for the CFI and whose psychometric properties have not been established for use with other instruments. However, given the preliminary nature and small sample size of this study, the quantitative scores should be interpreted as broad indicators of the overall evaluation and were intended to complement the qualitative findings. Future evaluations of the CIMI-M would benefit from more comprehensive psychometric assessment, including inter-interviewer reliability and content validity. Lastly, veterans identified as having experienced a PMIE and highly motivated clinicians with an interest in moral injury participated in the study. This may have introduced a positive bias, potentially leading to more favorable ratings of the CIMI-M. However, at this stage of interview development, administering the full interview required veterans with such experiences, while asking clinicians less familiar with moral injury to administer the interview would have risked conflating unfamiliarity with the construct with limitations of the instrument itself. The face-to-face debrief interview may also have introduced social desirability bias, potentially leading to more positive evaluations than participants might have provided in an anonymous format. Taken together, these findings reflect a preliminary evaluation of acceptability and feasibility within a selected, clinically relevant population and do not provide evidence of general feasibility across diverse settings and practitioners. For future evaluations of the interview, it would be valuable to include clinicians less familiar with moral injury, as well as veterans from more diverse backgrounds.

### Clinical implications

The CIMI-M facilitates the formulation of a comprehensive case conceptualization that encompasses all key elements relevant to moral injury, including a detailed description of the PMIE, an overview of the violated moral values, and an assessment of the impact of the PMIE across domains of moral injury, including coping strategies for dealing with moral pain. Systematically addressing these domains could aid clinicians in effectively matching therapeutic interventions to their patient’s needs. The case conceptualization can help identify, for instance, whether the patient requires interventions aimed at facilitating meaning-making or supporting social repair. Ultimately, tailoring treatment to the needs of the specific individual in this way may increase the likelihood of therapeutic success.

The CIMI-M was designed to complement existing intake procedures and should only be administered when patients report a PMIE, for example at intake. We recommend administering the MIOS [5] prior to the CIMI-M to screen for PMIE exposure, to determine whether the interview is warranted. The CIMI-M may also be used alongside PTSD assessments such as the CAPS-5 in cases when assessment of both PTSD and moral injury is deemed clinically relevant. Additionally, the CIMI-M’s explicit focus on violated moral values, PMIE attribution, and moral emotions may help clinicians distinguish morally injurious responses from PTSD and depression. Given the interview duration of at least 90 minutes, clinicians should carefully consider participant burden when combining multiple assessments. Importantly, however, clinicians anecdotally reported after the study that administering the CIMI-M prior to treatment saved time during treatment, as the CIMI-M already clarified violated moral beliefs and expectations, deepened veterans’ insight into their difficulties, and facilitated more targeted intervention selection. Moreover, while a minimum duration of 90 minutes may be considered lengthy in time-pressured clinical settings, providing veterans with sufficient time to share their experiences in depth was a central aim of the CIMI-M that was explicitly appreciated by veterans.

For successful implementation of the CIMI-M, lessons may be learned from the roll-out of the CFI [39]. Implementing a descriptive clinical interview with a person-centered, contextual approach rather than a symptom-oriented one, requires a fundamental shift in clinical assessment, particularly for clinicians trained in symptom-based diagnostics. As a result, the CFI was found to be at risk of becoming a mere checklist or of not being implemented at all [39]; challenges the CIMI-M might similarly face. To prevent the additional assessment being perceived as burdensome and to ensure the proper administration of the interview, training might be recommended for clinicians to make optimal use of the interview in clinical practice. We particularly recommend training for clinicians with limited familiarity with moral injury. For clinicians in the Netherlands, we developed two training videos in collaboration with the Netherlands Veterans Institute (each approximately 30 minutes), available online, covering the concept of moral injury and the administration of the interview. In-person training, as is common practice for the CAPS-5, may provide additional value by allowing clinicians to address questions around administration.

Finally, while the current version was developed for military veterans, adaptation for non-military populations would be needed for broader implementation. This would require modification of the questions about the military context. However, given the absence of other structured, evidence-informed treatment-planning instruments for moral injury, and given that the domains of impact underlying section 2 have demonstrated content validity among healthcare workers [40], we recommend that clinicians working outside military contexts consider using this section of the interview. This section is pivotal for treatment planning and may offer clinical value in non-military settings without requiring adaptation.

## Conclusion

In conclusion, the CIMI-M represents the first clinical interview specifically designed to assess moral injury. Its development involved the active engagement of various stakeholders and adhered to guidelines for intervention development, while also taking into account content validity. In a preliminary feasibility study, the CIMI-M was positively evaluated by veterans and clinicians and subsequently refined based on their feedback. Moving forward, we hope to adapt the CIMI-M for application in diverse populations affected by moral injury, such as healthcare workers, first responders, and refugees, thereby broadening its impact and relevance for the treatment of moral injury.

## Supporting information

S1 File Debrief interview questions. Questions used to debrief veterans and clinicians following administration of the CIMI-M as part of the feasibility study.
(DOCX)

S2 File Clinical Interview for Moral Injury – Military Version (CIMI-M). The full interview protocol of the CIMI-M, including instructions for administration, interview questions, and case conceptualization template.
(PDF)
