# Supplementary material for: Mapping moral injury: Development and feasibility of the Clinical Interview for Moral Injury—military version (CIMI-M)
Source: PLoS One. 2026 Sep 30;21(9):e0359553. doi: 10.1371/journal.pone.0359553 (PMC13626660; doi:10.1371/journal.pone.0359553)
Supplement: Supplementary file 1 [file pone.0359553.s002.pdf]

Clinical Interview for Moral Injury – Military version (CIMI-M)

Mariëlle L. de Goede [1,2], Paul A. Boelen [1,2], Brett T. Litz [3,4] & F. Jackie June ter Heide [1]

[1] ARQ Centrum'45, Diemen, the Netherlands; [2] Utrecht University, Utrecht, the Netherlands; [3] VA Boston Healthcare System, Boston, Massachusetts, USA; [4] Boston University and Boston University School of Medicine, Boston, Massachusetts, USA

CIMI-M version 1, January 2025. The latest version of the CIMI-M may be obtained from info@arq.org

Introduction

Purpose

The Clinical Interview for Moral Injury – Military version (CIMI-M) aims to determine whether a service member or veteran has experienced a potentially morally injurious experience (PMIE) and to assess its impact. Results from the CIMI-M can be used to create a case conceptualization and guide treatment planning. The CIMI-M can also be used to determine whether an interviewee is a clinical case, that is whether the interviewee experiences clinically significant problems after a potentially morally injurious experience that warrant treatment.

Structure

The CIMI-M has four sections. Section 1 assesses the military context in which the event(s) occurred. Section 2 assesses the potentially morally injurious experience(s). Section 3 explores the impact of these experiences across six domains of moral injury. Finally, section 4 examines the interviewee's desired change and readiness for change. A reporting template is provided at the end of the interview to summarize interview outcomes.

Potentially morally injurious experience

A PMIE is a stressor that may cause a unique form of distress because the event violates someone's moral beliefs and expectations. The violation of moral values and beliefs can result from *one's own* actions or *someone else's* behavior. Someone may violate their moral values by taking a particular action (an *act of commission*) or by not acting or failing to act (an *act of omission*). A PMIE can also entail events in which the person witnesses or is directly impacted by the transgressive behavior of others, such as enemy combatants, military leaders, or colleagues.

These events often carry serious consequences for the individual and/or others. While a PMIE may meet Criterion A for post-traumatic stress disorder (PTSD; namely, exposure to actual or threatened death, serious injury, or sexual assault), this is not always the case. For example, a service member who humiliated civilians in a war zone may experience moral distress without the event meeting Criterion A for PTSD. Thus, it is crucial to explore whether a person's deeply held beliefs about right and wrong were at stake during the event, regardless of its outcome (Bonson et al., 2023). A person may have felt strongly in the moment that what happened was "wrong," but may also have come to this realization later.

Moral injury

Moral injury is a potential clinical problem that can arise from experiencing one or more PMIEs. It typically involves clinically significant changes across six domains of functioning (Litz et al., 2022).

Domains of moral injury:

- Impairing moral emotions (guilt, shame, anger, disgust, etc.);
- Alterations in self- and other-perception (disruptions in how individuals define themselves or the world with respect to what they or others are capable of);
- Moral thinking (changes in moral thinking, such as condemning others on moral grounds or a hyper-focus on one's own moral behavior);
- Social impacts (changes in social behavior and the degree of connectedness with others, such as feeling socially isolated, difficulty trusting others or feeling empathy for others);
- Self-harming / self-sabotaging (conscious or unconscious behavior that negatively impacts one's health, safety, quality of life, functioning and happiness, such as poor self-care or suicidality);
- Beliefs about meaning and purpose (changes in ideas about the meaning and purpose of life, including religious, spiritual and existential beliefs).

Instructions for interview administration

Screening tools

It is recommended to use this interview when there is evidence that someone has experienced a PMIE, as indicated, for example, during an intake conversation. Before conducting the interview, it can be beneficial to also administer a questionnaire to screen for exposure to PMIEs, expressions of moral injury, and associated functional impairments, such as the *Moral Injury Outcome Scale* (MIOS; Litz et al., 2022). On the MIOS, endorsement of a PMIE coupled with a score between 26-31 suggests probable moral distress (sub-clinical but noteworthy moral injury), and a score equal or greater to 31 suggests the interviewee may experience clinically significant moral injury.

Interview duration

Administering the CIMI-M generally takes 90 to 120 minutes. Allow sufficient time, particularly for the questions about the PMIE and violated moral beliefs and expectations (section 2), and the questions about moral emotions and self- and other-perception (part of section 3). The latter part of the interview typically requires less time than the earlier sections.

Attitude

Discussing the PMIE(s) may bring out moral emotions and experiences (e.g., feelings of shame or anger and disgust) for the interviewee. For some, it may even be the first time sharing these experiences. It is therefore important to adopt a non-judgmental and supportive therapeutic attitude during the interview. If needed, remind the interviewee that sharing these experiences is understandably quite challenging. Reassure them that they only need to share what they feel comfortable sharing. Do not hesitate to ask follow-up questions, but prioritize creating a safe environment.

Guidance on questions

Not every interview question needs to be asked. The relevance of certain questions depends on the specific type of PMIE the interviewee has experienced. If a question is optional, this is indicated before the question.

Text that should be read to the interviewee (including questions and explanations) is italicized. Instructions for the interviewer are boxed and not italicized; this text is not meant to be read aloud. After some interview questions, you may see the following types of prompts:

- Follow-up questions: These are provided to help gather more information if the interviewee's initial response is limited.
- If unclear: These questions may be asked if the interviewee does not understand the original question.

Use the exact wording of the interview questions as much as possible. If needed, you can ask additional questions to clarify responses, but keep these to a minimum to limit the overall interview length.

Example

> ***2.3 What about this experience was the most distressing for you?***
>
> If unclear: *Did this experience go against your conscience?*
>
> Follow-up questions: *What about this experience felt unjust or wrong to you? In what way? Did this experience also conflict with your moral beliefs and expectations, or norms and values in other ways?*

Selecting the most distressing PMIE

If someone has experienced multiple PMIEs, the (impact of the) worst and currently most distressing PMIE is selected as the focus of the interview. If there's any uncertainty about whether an event qualifies as a PMIE, proceed with the interview to explore its impact further.

Questions that refer to the PMIE include the words '[the experience]'. We recommend using a term preferred or provided by the interviewee here.

Case conceptualization & treatment plan

The CIMI-M can be used to create an initial case conceptualization to aid in treatment planning. This case conceptualization can also be built upon or referred to during treatment. A template for case conceptualization is provided at the end of the interview.

When deciding if treatment for moral injury is appropriate, it is important that the person is experiencing clinically significant distress related to more than one domain of moral injury. The moral impact of an event can range in severity from *moral frustration* to *moral distress* and, at its most severe, moral injury (Litz & Kerig, 2019). Moral injury is characterized by clinically significant suffering. The clinical significance of a given domain is defined by the magnitude of the expressions of moral injury and the degree to which they negatively impact functioning.

For the treatment of moral injury several models exist. Most of these are cognitive-behavioral therapies and include treatments that focus on processing the morally injurious experience and reparative learning through virtuous and compassionate action (Adaptive Disclosure; Litz, 2024; Litz et al., 2017), challenging guilt-related cognitions[1] (TrIGR; Norman et al., 2019), or accepting tragic insights (ACT; Evans et al., 2020). Treatment should be selected based on the individual's needs, as not all approaches are equally relevant to a specific patient.

Patients may also have preferences regarding treatment format, such as individual versus group therapy, whether to involve their family and support network, and any additional care they would like, such as spiritual care (e.g., from a chaplain) or expressive therapy (e.g. creative therapy, music therapy or psychomotor therapy).

[1] During the interview, the interviewee may share guilt-related thoughts they experience since the PMIE. Guilt may stem from accurate appraisals of responsibility and culpability or be partly or fully caused by distorted beliefs and biased appraisals about culpability and responsibility (see Kubany et al., 1996; Norman et al., 2019).

Clinical Interview for Moral Injury – Military version (CIMI-M)

Name interviewee: ………………………………………… Date of birth: …………………………..……

Name interviewer: ………………………………………… Date: ……………………….……………

Introduction for the interviewee

<u>Introduction</u>: *The purpose of this interview is to explore if you have had a stressful experience in which you did or failed to do something that went against your values, conscience, or moral beliefs. Or you had an experience during which you witnessed others doing or failing to do things that went against your moral beliefs, or you were directly impacted by such behavior. Such an experience often feels extremely wrong, unjust, or unfair. Afterwards, you may have felt very ashamed, guilty, angry or disgusted by what happened. Experiences like these are called "morally injurious experiences". If you have been through such an experience, I would like to find out in what context this happened. I would also like to get a sense of the lasting impact of the experience. Do you understand what I mean by a morally injurious experience?*

<u>If the interviewee responds "no"</u>:

> *An example of a morally injurious experience could be a situation where doing "the right thing" was very important, but it was hard to determine what that was. Or, it might be a situation where you did or failed to do something that had serious consequences. It could also be something that someone else did that had serious consequences, and that affected you personally as a victim or witness. If it's still unclear what I mean by a "morally injurious experience," that's alright; we'll take time to explore together if you might have experienced something like this.*

If you have had a morally injurious experience, we can use this interview for planning your treatment, if necessary. Some of the questions may feel challenging, but you don't have to answer any question you're uncomfortable with—just let me know if you'd rather not answer a question.

In a moment I'll start with some general questions. If you need a short break during the interview, feel free to let me know. And please tell me if there's anything you don't understand.

Do you have any questions before we begin?

1. Context

Instruction: The following questions are intended to learn about the interviewee's norms, values, and moral expectations prior to the morally injurious event. Questions 1.4 and 1.5 can help clarify what the interviewee's actual responsibilities were at the time of the event. This may be helpful to keep in mind when asking question 3.3, which may bring up guilt-related thoughts. The interviewee may blame themselves for the event without actually having been responsible for it; this information may be relevant for treatment (see the instruction for question 3.3).

1.1 What motivated you to join the military?

………

………

………

………

………

………

………

………

1.2 Have you been deployed, and if so, where?

Follow-up questions: *Did you experience a potentially morally injurious experience during your deployment(s) or at another time? When were you deployed? How old were you then?*

………

………

………

………

…….

……

…….

…….

Instruction: Ask question 1.3 only if someone experienced a PMIE during their deployment.

1.3 What were you hoping to accomplish there?

If unclear: *What personal goal did you have when you went there? What expectations did you have?*

……

……

……

……

…….

……

……

…….

1.4 What were your role and rank during your deployment(s)?

……

……

……

……

……

……

……

……

1.5 For whom and what (specific tasks) where you responsible?

……

……

……

……

……

……

……

……

1.6 What were the most positive things that happened during your deployment(s) or in the service generally?

Follow-up questions: *Did you feel pride, a powerful sense of purpose and belonging to a group or unit? Or did you make friends for life, mature as a person, or did others have pride in you, like leaders, your family and friends, or the country?*

……

……

……

……

……

……

……

……

2. The potentially morally injurious experience(s)

Introduction: *I'm now going to ask you some questions about the morally injurious experience you may have had. So this entails an experience where something happened that went against your values, your conscience, or your ideas of right and wrong. If you've had multiple experiences like this, I'd like you to focus on the one that is currently the most distressing for you.*

2.1 Could you tell me what happened?

Follow-up questions: *When did this happen? Where were you? Who was involved? What happened, step-by-step? What did you do? What did others do? What were you feeling at the time? What were the consequences? How did others react?*

………

………

………

………

………

………

………

………

………

………

………

………

………

2.2 Have you had any other morally distressing experiences?

If yes: Explore these experiences in the same way.

Follow-up question: *Which of these experiences is currently causing you the most distress?*

………

………

………

………

………

………

………

………

………

………

………

………

………

………

………

………

………

………

Instruction: For this interview, focus on the experience that is currently the most distressing for interviewee. Select what type of PMIE this is:

PMIE type:

- ☐ The interviewee did something that violated their moral values (an *act of commission*);
- ☐ The interviewee failed to do something that violated their moral values (an *act of omission*). NB: this includes experiences where the interviewee tried to prevent the outcome but failed;
- ☐ The interviewee witnessed someone else violating their moral values or witnessed cruelty or the aftermath of mass violence;
- ☐ The interviewee was directly impacted by another person that violated the interviewee's moral values (e.g., someone betrayed their trust resulting in unnecessary danger or harm);
- ☐ The interviewee did not experience a PMIE.

If the interviewee did not experience a PMIE, the interview may be terminated.

2.3 What about this experience was the most distressing for you?

If unclear: *Did this experience go against your conscience?*

Follow-up questions: *What about this experience felt unjust or wrong to you? In what way? Did this experience also conflict with your moral beliefs and expectations, or norms and values in other ways?*

………

………

………

………

………

………

………

………

………

………

………

………

………

………

Instruction: Interviewees may find it challenging to articulate what their (violated) moral beliefs, expectations, or values are. Therefore, when posing question 2.3, it can be helpful to keep in mind the *foundations of morality*. According to the Moral Foundations Theory (Graham et al., 2013), there are five key foundations of morality, each associated with specific moral beliefs and values that most people share to some degree. These are:

**Foundation**	**Moral beliefs and values**
Care vs. harm	Moral beliefs: Others, particularly children and vulnerable people, should be protected and cared for.<br>Values: Caring, kindness.
Fairness vs. cheating	Moral beliefs: One should not lie or deceive others.<br>Values: Honesty, justice, trustworthiness, integrity.
Loyalty vs. betrayal	Moral beliefs: One should be loyal to the people or group to which one belongs.<br>Values: Loyalty, patriotism, self-sacrifice.
Authority vs. subversion	Moral beliefs: One should respect hierarchy and authority.<br>Values: Obedience, respect, deference.
Sanctity vs. degradation	Moral beliefs: One should respect what is sacred (e.g., life, the body, religious symbols).<br>Values: Reverence, respect, piety.

The importance placed on these moral beliefs and values may vary depending on the (sub)culture, social context, or individual.

3. Domains of moral injury

Domain of impact: impairing moral emotions

Instruction: This domain concerns predominant, pressing, and easily triggered moral emotions. Moral emotions include guilt, shame, remorse, humiliation, anger, disgust, or contempt.

3.1 How does this experience make you feel?

Follow-up questions (inquire about every emotion): *For example, do you feel guilt, shame, remorse, humiliation, anger, disgust, or contempt? Do you feel betrayed? Do you feel differently now compared to how you felt during or shortly after [the experience]? What has changed your feelings about [the experience] over time?*

……

……

……

……

……

……

……

……

3.2 How do you cope with these emotions?

Follow-up question: *What do you do when you are alone? How do you treat others? Does this behavior help you?*

……

……

……

………

………

………

………

………

Domain of impact: alterations in self- or other-perception

Instruction: This domain concerns disruptions in how individuals define themselves or the world with respect to what they or others are capable of. It entails negative alterations in ideas about personal or collective humanity. This is similar to the idea of shattered assumptions about a just world and personal invulnerability. Listen for themes of: seeing oneself as a bad person, loss of trust in one's ability to act morally or the ability of others to act morally, or feelings of self-hatred or inadequacy.

Instruction: Question 3.3 and 3.4 may not apply to every type of PMIE. Generally speaking, question 3.3 is appropriate to ask if the interviewee did or failed to do something that violated their values; question 3.4 is appropriate to ask if someone else did something that violated their values. However, in some cases both questions may be relevant. Assess the relevance of these questions before including them in the interview.

3.3 How have your thoughts and beliefs about yourself changed as a result of [the experience]?

If unclear: *Do you think differently about the extent to which you are a good person or can be trusted?*

Follow-up questions: *Do you feel like you are a bad person? Do you struggle to forgive yourself? If so, what is unforgivable? Has [the experience] affected your self-image, self-worth, or self-confidence in other ways?*

………

………

………

………

……………………………………………………………………………………………………….

………………………………………………………………………………………………………

………………………………………………………………………………………………………

………………………………………………………………………………………………………

3.4 How have your thoughts and beliefs about other people or the world changed as a result of [the experience]?

If unclear: *Do you think differently about the extent to which people can be good and the world is a good and just place?*

Follow-up questions: *Do you feel like others can't be trusted and if you don't look out they will fail you? Do you struggle to forgive others for anything? If so, what is unforgivable? Has [the experience] affected your faith in other people in other ways?*

………………………………………………………………………………………………………

………………………………………………………………………………………………………

………………………………………………………………………………………………………

………………………………………………………………………………………………………

……………………………………………………………………………………………………….

………………………………………………………………………………………………………

………………………………………………………………………………………………………

………………………………………………………………………………………………………

Instruction: Question 3.5 explores whether the interviewee has experienced other potentially traumatic events that may shape how they view the PMIE. Such events might include adverse childhood experiences and other incidents that contribute to negative core beliefs like "I am a bad person" or "others cannot be trusted". If these beliefs were reinforced during the morally injurious experience, it can intensify its impact and make recovery more challenging.

3.5 Did you have any earlier experiences in life that influence how you perceive [the experience]?

If yes: *In what ways?*

………

………

………

………

………

………

………

………

Domain of impact: moral thinking

Instruction: This domain concerns changes in moral thinking. It entails a person judging themselves or others harshly, moralistically, and with condemnation. Listen for themes of: critical thoughts about oneself or others, a hyperfocus on moral behavior, and constant and deliberate thinking about the morally injurious experience.

3.6 Do you have a tendency to judge or condemn yourself or others harshly, since [the experience]?

Follow-up questions: *In what ways?*

………

……

……

……

……

……

……

……

Instruction: Question 3.7 may not apply to every type of PMIE; assess its relevance before including it in the interview. It is important here to distinguish between *deliberate* thinking and intrusive thoughts. Ensure that the interviewee is intentionally thinking about the experience, rather than experiencing uncontrollable intrusions.

3.7 Do you feel like you need to keep thinking about [the experience] or the people involved, because you can't allow yourself to forget them or what happened?

If yes: *In what ways?*

……

……

……

……

……

……

……

……

Instruction: Ask question 3.8 only if *someone else* did or failed to do something that went against the interviewee's moral beliefs.

3.8 Do you think or have fantasies about taking revenge on people who were involved in [the experience]?

If yes: *In what ways?*

……

……

……

……

……

……

……

……

Domain of impact: self-harming and self-sabotaging

Instruction: This domain concerns conscious and unconscious behaviors that negatively impact the interviewee's functioning, health, safety, and quality of life. Listen for themes of: lack of self-care or motivation, self-sabotaging, substance abuse, engaging in risky or aggressive behaviors, self-harming, or suicidality.

3.9 Do you take less good care of yourself or your health, because of [the experience]?

If yes: *In what ways?*

……

……

……

………

………

………

………

………

3.10 Do you undermine or sabotage yourself, because of [the experience]?

<u>If yes:</u> *In what ways?*

<u>If unclear</u>: *Do you feel like you no longer deserve to succeed, experience positive feelings, or enjoy life?*

………

………

………

………

………

………

………

………

3.11 Do you engage in aggressive or risky behaviors, because of [the experience]?

<u>If yes:</u> *In what ways?*

………

………

………

………

………

………

………

………

3.12 Do you numb your feelings or harm yourself, because of [the experience]?

If yes: *In what ways?*

If unclear: *Do you use alcohol or other substances, or do you harm yourself physically, to cope with the emotional pain?*

………

………

………

………

………

………

………

………

Instruction: If the interviewee mentions suicidal thoughts in response to question 3.13, inquire about the nature of these thoughts and concrete plans. If the interviewee has concrete plans to end their life, follow your institution's protocol for handling suicidal behavior and/or ask the interviewee's permission to inform their primary therapist.

3.13 Do you ever think about ending your life, because of [the experience]?

If yes: *Can you tell me a little more about those thoughts?*

……

……

……

……

……

……

……

……

Domain of impact: social impacts

Instruction: This domain concerns changes in social behavior and the degree of connectedness with others. Listen for themes of: lack of interest in social relationships, decreased trust in others, loss of community, social isolation, difficulty feeling empathy for others, or expecting rejection and judgement.

3.14 If you have a partner and/or children, does [the experience] affect your relationship with your partner and/or child(ren)?

If yes: *In what ways?*

Follow-up questions: *Do you have difficulty trusting them or sharing how you feel? Do you tend to isolate yourself from them? Do you have difficulty feeling empathy for them? How do they see [the experience], or how would they see it if they knew about it? How does this affect you?*

………

………

………

………

………

………

………

………

3.15 Does [the experience] affect other important relationships in your life, such as your relationships with friends, colleagues, or the community to which you belong?

If yes: *In what ways?*

Follow-up questions: *Do you have difficulty trusting them or sharing how you feel? Do you tend to isolate yourself from them? Do you have difficulty feeling empathy for them? How do they see [the experience], or how would they see it if they knew about it? How does this affect you?*

………

………

………

………

………

………

………

………

Domain of impact: beliefs about meaning and purpose

Instruction: This domain concerns changes in a person's beliefs about meaning and purpose, including religious, spiritual, or existential ideas. Listen for themes of: loss of (or a negatively altered relationship with) religion or spirituality, loss of belief in the meaning of life, or loss of the belief that a human life is valuable.

3.16 Does [the experience] affect the way you look at life? Think for example about how you view the meaning of life or the value of human life.

If yes: *In what ways?*

………

………

………

………

………

………

………

………

3.17 Do you see yourself as a religious or spiritual person? If so, does [the experience] affect your faith or spirituality?

If yes: *In what ways?*

Follow-up questions: *Do you currently find strength in your faith or spirituality? In what ways?*

………

………

………

………

………

………

………

………

Impact on other areas of life

> Instruction: Use question 3.18 to explore other areas of functioning that have not yet been addressed during the interview, if relevant (e.g., ask about the interviewee's sexual functioning if the morally injurious event involved sexual violence).

3.18 Does [the experience] affect your daily functioning in other ways?

Follow-up question: *What isn't going well? What is going well?*

Follow-up questions [if relevant]: *Does [the experience] affect your daily activities or hobbies? Does [the experience] affect your work? Does [the experience] affect your physical health? Does [the experience] affect how you experience your sexuality?*

………

………

………

………

………

………

………

………

4. Desired change and readiness for change

4.1 Of all the ways [the experience] affects your life, what would you most like to change or need help with?

Follow-up question: *If you make changes in this area, what would be different in your life?*

………

………

………

………

………

………

………

………

4.2 Have you tried working on these problems yourself?

If yes: *What have you tried? What worked? What didn't? Why do you think that is?*

………

………

………

………

………

………

………

…….

<u>Instruction</u>: Use question 4.3 to discuss treatment options with the interviewee (e.g., trauma-focused therapy, individual or group treatment, online interventions, The Moral Injury Workbook, etc.).

4.3 What kind of help do you think you need?

<u>Follow-up questions</u>: *Do you prefer individual or group treatment? Is it important for you to involve your family, loved ones, or your community? Is it important for you to include your faith or spirituality? What would you recommend to someone going through a similar experience?*

……

……

……

……

…….

……

……

…….

4.4 Is there anything else you would like to add or discuss?

……

……

……

……

……

……

……

……

Reporting template

Date: ....

Name interviewee: ....

Name interviewer: ....

Case conceptualization

Use the space below to brainstorm and hypothesize about the causes of the person's unique constellation of problems, and in particular the modifiable characteristics that might serve as targets for treatment. This is an initial descriptive functional case conceptualization. This can serve as a foundation for creating a more detailed, explanatory case conceptualization in collaboration with the patient during treatment. For example, using this case conceptualization, you might explore the function of coping behaviors (see Borges et al., 2022).

It might help to bear in mind the following overview (taken from Litz & Walker, in press):

MI subtype	Others' behavioral, emotional, and belief-based reactions	Behavioral, emotional, and belief-based signs of harm	What is potentially lost?	Recovering what is lost by leveraging / creating corrective learning opportunities in therapy, at home, at work, in communities
**Shame-related phenomena**	Anger, fear, empathy failure, bitterness, disowning, exclusion/rejection, condemnation, fundamental attribution bias (the event defines the transgressor; it is a trait)	Submission, avoidance of intimacy and scrutiny, isolation, anxiety from loss of social standing, dysphoria, anhedonia, self-dehumanization, desire for the community to know that the event does not define the person (unless the person believes the event is self-defining)	Pride, value/self-worth, self-compassion, social cohesion, prosocial relationships, belongingness, humanity, the balance of personal goodness, relative to badness, comfort and safety, deservedness, hedonic relational experiences	Confess/disclose the harm, share/express impact, get compassionate validation; clarify meaning / implication; get feedback from caring others (and professionals) about the implication of the event and help generating a repair plan<br>Daily purposeful virtuous behaviors, especially compassion, in ad hoc and planned ways<br>Mindful allowing of others to show their humanity, be kind, trustworthy, and selfless
**Trust-violation-related phenomena**	Fear, helplessness, frustration, empathy failure, disconnection, disbelieving, fundamental attribution bias (the person is angry and bitter)	Broken heartedness, anger, disillusionment, intolerance, grievance, outrage, cynicism, indifference, loss of faith in humanity, authorities, and institutions, social vigilance and distrust, justice-seeking, confirmation-seeking, demoralization	Social comfort and cohesion, prosocial relationships, trust/faith in humanity, belongingness, compassion, optimism, beliefs/expectancies about mattering, justice, the potential for good in others, hedonic relational experiences.	Working on belonging: Increasing the size and reach of "Us" groups and reducing the size and reach of "Other" groups<br>Doing valued things, being a part of groups that are valuing<br>Taking misdeeds, missteps, unempathetic, rejecting, and selfish behaviors by self and others in stride - developing perspective and tolerance<br>Generating a maintenance plan and planning response to setbacks

Military service and deployment context

E.g., what positive repertoires and beliefs have been altered and lost because of the morally injurious experience? To what extent has the interviewee lost personal or collective value and kinship (belonging) and to what extent might these things be reclaimed in other ways to repair the experience?

…….

…….

…….

…….

…….

The potentially morally injurious experience

E.g., what happened? Was the event singular in nature or did it happen repeatedly? Can the experience be tied thematically to shame- or trust-violation problems, or both?

…….

…….

…….

…….

…….

PMIE type:

- ☐ The interviewee did something that violated their moral values (an *act of commission*);
- ☐ The interviewee failed to do something that violated their moral values (an *act of omission*). NB: this includes experiences where the interviewee tried to prevent the outcome but failed;
- ☐ The interviewee witnessed someone else violating their moral values or witnessed cruelty or the aftermath of mass violence;
- ☐ The interviewee was directly impacted by another person that violated the interviewee's moral values (e.g., someone betrayed their trust resulting in unnecessary danger or harm).

Violated moral beliefs and expectations

E.g. what moral beliefs and expectations were violated or played a key role during the PMIE?

……

……

……

……

……

Domain of impact: impairing moral emotions

E.g., what appears to trigger moral emotions? What are the precipitating events and consequences?

……

……

……

……

……

Domain of impact: self- and other-perception

E.g., how much has the interviewee lost faith in their own humanity or in humanity? What might help the interviewee repair the loss of faith in the goodness of the self or others?

……

……

……

……

………

Domain of impact: moral thinking

E.g., how pressing and impairing is this problem? What might help the interviewee be less moralistic and judgmental in their lives currently?

………

………

………

………

………

Domain of impact: self-harming and self-sabotaging

E.g., how pressing and impairing are these problems? To what extent might these problems change if the interviewee engaged in reparative activities socially and in terms of leisure? Would it help to do good and be compassionate, or to allow others to be good and caring?

………

………

………

………

………

Domain of impact: social impacts

E.g., to what extent is the interviewee socially isolated and avoidant of social activity that would otherwise provide corrective and reparative moral experiences? E.g. doing good and being compassionate to others as a way of addressing self-hatred, allowing others to be good, trusting others however cautiously to restore faith in humanity, etc.

……………………………………………………………………………………………………….

……………………………………………………………………………………………………….

……………………………………………………………………………………………………….

……………………………………………………………………………………………………….

……………………………………………………………………………………………………….

Domain of impact: beliefs about meaning and purpose

E.g., how might connections with others, belonging to a community, and other activities shift existential harm?

……………………………………………………………………………………………………….

……………………………………………………………………………………………………….

……………………………………………………………………………………………………….

……………………………………………………………………………………………………….

……………………………………………………………………………………………………….

Impact on other areas of life

……………………………………………………………………………………………………….

……………………………………………………………………………………………………….

……………………………………………………………………………………………………….

……………………………………………………………………………………………………….

……………………………………………………………………………………………………….

Desired change and readiness for change

……

……

……

……

……

Caseness

Determine caseness (i.e. does the interviewee most likely experience clinically significant moral injury that warrants treatment) by answering the following questions:

1) Has the interviewee experienced a high magnitude and currently distressing PMIE?
2) Is the PMIE associated with current functionally interfering problems within more than one domain of impact?
3) Is there sufficient information to suggest that these problems are new onset (i.e. began or worsened after experiencing the PMIE)?
4) Is there sufficient evidence that recovery has been impeded or not likely and moral injury should be considered a pressing problem that should be addressed clinically?

Treatment options

Based on the interview findings and patient preferences, indicate the most appropriate treatment approach. Multiple options can be selected.

Treatment focus:

- ☐ Trauma-focused therapy (to reduce intrusions or intense emotional pain);
- ☐ Challenging guilt-related thoughts or self-blame (distortions regarding one's own role and responsibility in the event);
- ☐ Meaning-making (working on negative perceptions of self and/or others);
- ☐ Social recovery (living according to one's own moral values within one's community).

Treatment format:

- ☐ Individual therapy
- ☐ Group therapy

Involvement of support system:

- ☐ No
- ☐ Yes: ………………………………………………………………………………………………………..

Additional care:

- ☐ Spiritual care/chaplain
- ☐ Expressive arts therapist
- ☐ Other: ……………………………………………………………………………………………………

Summary

On … (date), the Clinical Interview for Moral Injury – Military version (CIMI-M) was conducted with … (name). The patient concerns ... (gender, age, target population, deployment).

Experience(s)

The CIMI-M revealed *no exposure to a morally injurious experience* / *exposure to* (choose: *a moral challenge / moral stressor / potentially morally injurious experience*), namely … (describe the event). During this event, (select: *the patient / another person*) (select: *did / failed to do*) something that violated the patient's moral values. This event can be categorized as (describe PMIE type, e.g. *an act of commission / omission,* etc.). The violated values pertain to ... (specify values).

Impact

As a result, the patient reports difficulties across multiple domains, including (choose: *impairing moral emotions / self- and other-perception / moral thinking / self-harming and self-sabotaging / social impacts / beliefs about meaning and purpose*). Regarding impairing moral emotions, the patient experiences ... (specify). Changes in self- and other-perception are evident in ... (specify). Regarding moral thinking, the patient reports ... (specify). Dysfunctional coping mechanisms include ... (specify). The social impacts concern... (specify). Alterations in beliefs about meaning and purpose are evident in ... (specify). Additional impact on other areas of life includes ... (specify). The overall impact can be characterized as (choose: *moral frustration / moral distress / moral injury*).

Recommendations for treatment

The recommended approach includes (select: *no treatment / psychoeducation / treatment focused on* ... specify focus, e.g., *trauma-focused therapy / addressing guilt-related cognitions / meaning-making / social recovery*). The patient has expressed a preference for (select: *individual therapy / group therapy*) with (select: *system involvement / no system involvement*). If system involvement is preferred, this involves: ... (specify details). Additional care is recommended, including (select: *spiritual care / expressive therapy / other: …*).

How to cite this interview:

De Goede, M.L., Boelen, P.A., Litz. B.T., & Ter Heide, F.J.J. (2024). *Clinical interview for moral injury – Military version (CIMI-M)*. Diemen: ARQ National Psychotrauma Centre.

References

Borges, L. M., Barnes, S. M., Farnsworth, J. K., Drescher, K. D., & Walser, R. D. (2022). Case conceptualizing in acceptance and commitment therapy for moral injury: An active and ongoing approach to understanding and intervening on moral injury. *Frontiers in Psychiatry, 13,* 910414. https://doi.org/10.3389/fpsyt.2022.910414

Bonson, A., Murphy, D., Aldridge, V., Greenberg, N., & Williamson, V. (2023). Conceptualization of moral injury: A socio-cognitive perspective. *Journal of Military, Veteran and Family Health*, *9*(2), 75-81. https://doi.org/10.3138/jmvfh-2022-0034

Evans, W. R., Walser, R. D., Drescher, K. D., & Farnsworth, J. K. (2020). *The moral injury workbook: Acceptance and commitment therapy skills for moving beyond shame, anger, and trauma to reclaim your values.* New Harbinger Publications.

Graham, J., Haidt, J., Koleva, S., Motyl, M., Iyer, R., Wojcik, S. P., & Ditto, P. H. (2013). Moral foundations theory: The pragmatic validity of moral pluralism. In P. Devine & A. Plant (Eds.), *Advances in experimental social psychology* (Vol. 47, pp. 55-130). Academic Press. http://dx.doi.org/10.1016/B978-0-12-407236-7.00002-4

Kubany, E. S., Haynes, S. N., Abueg, F. R., Manke, F. P., Brennan, J. M., & Stahura, C. (1996). Development and validation of the trauma-related guilt inventory (TRGI). *Psychological Assessment*, *8(4)*, 428. httpsL//doi.org/10.1037/1040-3590.8.4.428

Litz, B. T. (2024). A functional approach to defining and repairing moral injury: Evidence, change agents, clinical strategies, and lessons learned. *Journal of Traumatic Stress*, 37(5), 775-783. https://doi.org/10.1002/jts.23063

Litz, B. T., & Kerig, P. K. (2019). Introduction to the special issue on moral injury: Conceptual challenges, methodological issues, and clinical applications. *Journal of Traumatic Stress*, 32(3), 341-349.

Litz, B. T., Lebowitz, L., Gray, M. J., & Nash, W. P. (2017). *Adaptive disclosure: A new treatment for military trauma, loss, and moral injury.* Guilford Publications.

Litz, B. T., Plouffe, R. A., Nazarov, A., Murphy, D., Phelps, A., Coady, A., ... & Moral Injury Outcome Scale Consortium. (2022). Defining and assessing the syndrome of moral injury: Initial findings of the moral injury outcome scale consortium. *Frontiers in psychiatry, 13,* 923928. https://doi.org/10.3389/fpsyt.2022.923928

Litz, B. T., Stein, N., Delaney, E., Lebowitz, L., Nash, W. P., Silva, C., & Maguen, S. (2009). Moral injury and moral repair in war veterans: A preliminary model and intervention strategy. *Clinical psychology review, 29(8),* 695-706. https://doi.org/10.1016/j.cpr.2009.07.003

Litz, B. T., & Walker, H. E. (in press). Moral injury: An overview of conceptual, definitional, assessment, and treatment issues. *Annual Review of Clinical Psychology*.

Norman, S., Allard, C., Browne, K., Capone, C., Davis, B., & Kubany, E. (2019). *Trauma informed guilt reduction therapy: Treating guilt and shame resulting from trauma and moral injury*. Academic Press.
