# Supplementary material for: Mapping moral injury: Development and feasibility of the Clinical Interview for Moral Injury—military version (CIMI-M)
Source: PLoS One. 2026 Sep 30;21(9):e0359553. doi: 10.1371/journal.pone.0359553 (PMC13626660; doi:10.1371/journal.pone.0359553)
Supplement: Supplementary file 2 [file pone.0359553.s001.docx]

*Supplement 1: Debrief interview questions*

Debrief questions for patients

1. What did you think of the interview overall?
2. How would you evaluate the interview on a scale from 0 to 10 and can you explain why?
3. If you had to give at least one suggestion to improve the interview, what would it be?
4. What did you think of the structure of the interview?
5. What did you think of the length of the interview?
6. Did this interview help you share your experience(s), and if so, how?
7. What did you find the most helpful question during this interview? What did you find the least helpful question?
8. Were there any questions that you found difficult to answer? Why was that?
9. Are there any questions in the interview that you think should be changed or removed, for example, because they were unclear? Are there any questions that were not asked that you think should have been asked?
10. How do you think the interview will affect your treatment?
11. Do you have any other suggestions to improve the interview?

Debrief questions for clinicians

1. What did you think of the interview overall?
2. How would you evaluate the interview on a scale from 0 to 10 and can you explain why?
3. If you had to give at least one suggestion to improve the interview, what would it be?
4. What did you think of the structure of the interview?
5. What did you think of the length of the interview?
6. Did the interview help you obtain important information that you might not otherwise have asked about, and if so, how?
7. Did the interview affect the therapeutic relationship with the patient, and if so, how?
8. What did you find the most helpful question during this interview? What did you find the least helpful question?
9. Are there any questions in the interview that you think should be changed or removed, for example, because they were unclear? Are there any questions that were not asked that you think should have been asked?
10. How and when do you think this interview is best conducted? Do you think the interview will help determine caseness and create a treatment plan?
11. Do you have any other suggestions to improve the interview?
