# Supplementary material for: Mapping moral injury: Development and feasibility of the Clinical Interview for Moral Injury—military version (CIMI-M)
Source: PLoS One. 2026 Sep 30;21(9):e0359553. doi: 10.1371/journal.pone.0359553 (PMC13626660; doi:10.1371/journal.pone.0359553)
Supplement: Supplementary file 4 [file pone.0359553.s005.docx]

*Dear Editor and reviewer,*

*Thank you for the opportunity to further revise and resubmit our manuscript ‘Mapping moral injury: Development and feasibility of the Clinical Interview for Moral Injury—Military Version (CIMI-M).’ We are grateful to the reviewer for the thoughtful comments. We have adapted the manuscript according to your suggestions; please see our responses below.*

Reviewer #3: Thank you for the opportunity to review the manuscript titled Mapping moral injury: Development and feasibility of the Clinical Interview for Moral Injury—Military Version (CIMI-M) submitted to PLOSOne. I do not believe I was a reviewer on the previous version of the manuscript, so my initial review will be based on the most updated submission.

This reflects an important initiative as moral injury is historically understudied and its investigation outside of military populations is essentially in its infancy. However, there are several notable concerns that I highlight below that I believe will substantively enhance the contribution of this work to the greater psychological literature.

1. Page 2, first paragraph - this paragraph seems to end more abruptly with a brief description of moral injury-related debates, but this section seems to trail off. I recommend ensuring this section is more cohesive.

*We appreciate that this reviewer recognizes the value of our work and thank them for this observation. We agree that the paragraph ended somewhat abruptly and have revised it to improve cohesion. Specifically, we added a closing sentence that connects the ongoing conceptual debates to the need for descriptive assessment tools: “These unresolved have direct implications for how moral injury is assessed clinically, and underscore the need for flexible, descriptive assessment tools that are agnostic about exposure requirements and diagnostic boundaries.”*

2. 'Current moral injury questionnaires, for instance, do not inquire about what moral beliefs were violated, which is relevant for treatment.' This is not true, at least for the Moral Injury Outcome Scale. The first set of items prior to the outcome items reflect the nature of the trauma.

*The Moral Injury Outcome Scale inquires about the nature of the potentially morally injurious experience (whether it involved perpetration, witnessing, or betrayal). However, the claim we intend to make is that existing questionnaires do not inquire about which specific moral beliefs or values were violated—for instance, values such as care, fairness, or loyalty. We believe this information is directly relevant for treatment planning. We have revised the sentence in the manuscript to make this explicit: “Current moral injury questionnaires, for instance, do not inquire about what specific moral beliefs or values were violated (such as care, fairness, or loyalty), which is relevant for treatment planning.”*

3. The authors note that a diagnostic clinical interview is not feasible at this stage of construct investigation, which I do not disagree with. However, it is worth examining the following reference to understand and integrate any relevant diagnotic cut-offs that have been proposed (perhaps integration into the Instrument Aim subsection would also be helpful): Litz, B. T., Walker, H. E., Rusowicz-Orazem, L., Styler, Z. R., Fielstein, E., Darnell, B., ... & Nieuwsma, J. A. (2026). Establishing Clinically Significant Change Benchmarks for the Moral Injury Outcome Scale in VA Behavioral Health Settings. Assessment, 10731911261436687.

*A similar concern was raised by a reviewer in the previous round of review, to which we responded as follows: although research has identified severity score thresholds on moral injury questionnaires, moral injury does not currently have a configural diagnostic decision rule nor is it codified as a mental disease. The purpose of the CIMI-M is to guide case formulation and treatment planning, which would be valuable even if a clinical interview analogous to the CAPS-5 existed that determined a formal diagnosis of moral injury. At this point in the clinical science of moral injury, if clinicians want to identify probable clinical caseness as a gatekeeping function for initiating a CIMI-M, they can use the MIOS, which for veterans has a caseness threshold of >31.*

*We hope that the reviewer can appreciate this argument. The CIMI-M was deliberately designed as a descriptive tool rather than diagnostic instrument. If there was a valid configural way to determine moral injury caseness, the CIMI-M could easily be modified as a diagnostic and treatment planning interview. But this is not the current state of the field. Users may wish to use severity score probable caseness as a gatekeeping function prior to administering the CIMI-M, which is time intensive. However, this is not something we would recommend because of the possibility of false negatives among treatment-seeking service members and veterans. Also, true non-cases may have moral injury symptoms and related impairments that are potential treatment targets because they may affect motivation and compliance or collaterally impact a primary presenting problem.*

4. Have the authors considered the different populations for which moral injury may be a relevant construct? For example, would there be different presentations and/or interview questions for Veterans versus first responders versus health care workers in general versus civilians, etc? This may be worth describing.

*We agree with the reviewer that different populations require tailored interview questions. This is addressed in the manuscript in several sections:*

*In the limitations section, we note that the context-related questions, which inquire about the interviewee's deployment(s) and reasons for joining the military, would require modification if the CIMI-M were to be adapted for civilians or individuals in other professions. We also note in the clinical implications section that adaptation for non-military populations would be needed for broader implementation, and that this would require modification of the questions about the military context and, potentially, the domains of impact (as the study that established the moral injury domains relied on data from military populations exclusively). In the conclusion, we explicitly refer to healthcare workers, first responders, and refugees as populations for whom we hope to adapt the CIMI-M in future work.

Finally, upon reflection, because there is no other structured evidence-informed treatment-planning instrument in the field and because the MIOS, which established the content validity of the dimensions of impact used in the CIMI-M, has been validated in several studies of healthcare workers (e.g., Plouffe et al., 2025), we recommend that clinicians outside military and veteran contexts consider using section 2 of the CIMI-M. Section 2 asks respondents about domains of impact and this section is pivotal for treatment planning. We have added this recommendation in the discussion section:
“However, given the absence of other structured, evidence-informed treatment-planning instruments for moral injury, and given that the domains of impact underlying section 2 have demonstrated content validity among healthcare workers [40], we recommend that clinicians working outside military contexts consider using this section of the interview. This section is pivotal for treatment planning and may offer clinical value in non-military settings without requiring adaptation.”*

*Reference: Plouffe, R. A., Houle, S. A., Birch, M., Ein, N., Nazarov, A., & Richardson, J. D. (2025). Validation of the Moral Injury Outcome Scale in Canadian health care workers. Psychological Assessment, 37(6–7), 309–321. https://doi.org/10.1037/pas0001386*

5. Could the authors describe at the outset (i.e., in the measure development section) how the interview questions differ from those developed for relevant self-report measures of moral injury?

*We believe this comment, along with several others in this review, may partly reflect the fact that the full CIMI-M was not made available to the reviewer as a supplement. The CIMI-M was submitted in full as S2 Supporting Information, and we believe that access to this document would have substantially clarified how the interview questions differ from existing self-report measures. We hope the reviewer will have the opportunity to consult this supplement alongside the revised manuscript.*

*To address the question: the CIMI-M differs from existing self-report moral injury measures in several fundamental ways. Existing self-report instruments consist of closed-ended items rated on Likert scales, which ask respondents to quantify the frequency or severity of specific experiences or outcomes. By contrast, the CIMI-M uses open-ended* real-time interview questions *designed to elicit narrative responses, allowing veterans to describe their experiences in their own words and enabling the clinician to probe for depth and clarify ambiguities. The aim is not to generate a severity score. Instead, the CIMI-M was designed to generate qualitative clinical information about the nature of the PMIE, the specific moral values that were violated, the meaning attributed to the event, and the domains of impact that are most pressing. This information is relevant to case conceptualization and treatment planning. Existing self-report questionnaires are not designed for this purpose.*

6. Did the authors consider any moral distress literature, and how were the two constructs differentiated for development of this particular interview?

*The CIMI-M was developed primarily within the military moral injury literature, which uses the term “moral distress” to refer to subthreshold responses to PMIEs along a continuum of severity, rather than in the sense used in the healthcare and nursing literature, where moral distress typically refers to the psychosocial and occupational burden of being under resourced and overworked or having poor leadership, or doing things or being prevented from acting in accordance with one's ethical obligations in an institutional context. While these constructs overlap conceptually, they address different populations and are operationalized differently. The CIMI-M was developed specifically for military veterans and its theoretical foundations draw on the moral injury frameworks of Litz et al. [1, 5] and Farnsworth et al. [34]. Therefore, the healthcare moral distress literature was not the primary reference point for its development. We agree, however, that if the CIMI-M is adapted for non-military populations such as healthcare workers, engagement with that literature will be relevant.*

7. Example question listed on p. 10 is a closed-ended question, which generally are not as informative in qualitative research. Were most questions like this? Was this considered in their development? Were specific probing questions developed? Why/why not?

*We thank the reviewer for this observation and again note that access to the full CIMI-M as S2 Supporting Information would have provided important context for this comment. The majority of questions in the CIMI-M are open-ended; however, some are closed questions. This is because some questions address behaviors that not all veterans will show, for example: “Do you undermine or sabotage yourself, as a result of [the experience]?” Phrasing this as an open question ("How do you undermine or sabotage yourself...") would presuppose that the behavior is present, which could feel invalidating to veterans for whom it is not. A closed question first establishes whether the experience is relevant to the individual, before inviting elaboration.*

*Furthermore, each closed question in the CIMI-M is accompanied by specific follow-up questions to elicit more details when the respondent answers affirmatively. In this case, that follow-up question is: “In what ways?”*

*Interestingly, the feasibility study revealed that even when questions were phrased as closed questions, most veterans spontaneously elaborated on their answers rather than simply responding “yes” or “no,” suggesting that the interview format naturally facilitated open dialogue even when asking closed questions.*

8. There is not a lot of information provided about the theoretical development or background on moral injury; this almost seems tangential to the rest of the paper. I think it would strengthen the manuscript considerably to focus more on the theoretical foundations underlying this work (more than a brief overview).

*We thank the reviewer for this suggestion. We respectfully note, however, that the primary aim of this manuscript is to describe the development and preliminary feasibility evaluation of the CIMI-M, rather than to reflect upon the theoretical foundations of moral injury. As such, a comprehensive review is beyond the scope of this paper, and expanding this section substantially would add considerable length to a manuscript that is already quite comprehensive. We would direct interested readers to Litz and Walker (2025) [9] for a thorough overview of conceptual, definitional, and theoretical issues in moral injury.*

*That said, we take the reviewer's point that the connection between the theoretical foundations and the interview content could be made more explicit. We have therefore added the following sentence to the theoretical framework section of the manuscript, to clarify how each theory informed specific aspects of the CIMI-M: “In the final interview, each theory informed distinct components of the interview: the working model of Litz et al. [1] underpins the questions about attributions made in relation to the PMIE; the functional approach of Farnsworth et al. [34] informed questions about coping with moral pain and self-harming or self-sabotaging behaviors; and the six-domain framework of Litz et al. [5] provided the organizing structure for the impact section of the interview.”*

9. Qualitative methodology (e.g., how themes were decided upon and extracted) could be more detailed.

*Following this suggestion, we have expanded the description of the qualitative methodology in the Methods section to provide greater detail about how themes were identified and extracted. This section now reads as follows: “Answers to the debrief interview were inserted in MAXQDA 2022 and analyzed by the first author using the General Inductive Approach for coding qualitative evaluation data [37]. Analysis proceeded in four steps. First, all text segments relevant to the evaluation objectives were identified and marked. Second, relevant segments were labeled to create an initial set of categories, producing 27 codes. Third, codes were reviewed and merged to minimize redundancy and overlap, resulting in 10 labels. For example, the codes ‘reflection on experiences’, ‘awareness of the impact’, and ‘new insights’ were merged into the label ‘reflection and insight’. Fourth, we grouped these labels into overarching categories reflecting the evaluation objectives of feasibility, acceptability, and clinical utility. The evaluation objectives guided the focus of the analysis without imposing a priori expectations about specific findings, consistent with the General Inductive Approach [37].”*

10. 'On the DIP, clinical utility was rated M=3.34 (SD=0.47, range 2-4), feasibility M=3.39 (SD=0.55, range 2-4).' Can the authors please define clinical utility, feasibility and acceptability for unfamiliar readers?

*Following your suggestion, we added brief definitions of these three constructs to the manuscript, at the point where they are first introduced. The following sentence has been added to the Methods section, under the header ‘Design’: “Feasibility refers to the extent to which the interview can be practically administered in the intended clinical context; acceptability refers to the degree to which the interview is experienced as appropriate, reasonable, and tolerable by both veterans and clinicians; and clinical utility refers to the extent to which the interview generates information that supports clinical decision-making, case conceptualization, and treatment planning.”*

11. When the authors state that Veterans and clinicians were recruited, were the Veterans recruited to take part in the interview and clinicians recruited to deliver the interview to these participants? Please be specific. Were those who conducted the interview adequately prepared and trained in a systematic way? What were participants' gender and ages? Please provide details.

*Regarding recruitment: veterans were recruited to take part in the CIMI-M as interviewees, and clinicians were recruited to administer the interview to these participants. Where a veteran was already being seen by a clinician sufficiently familiar with the concept of moral injury, that clinician was asked to administer the CIMI-M. Where no such clinician was already involved in the veteran's care, a clinician with relevant familiarity was approached separately to conduct the interview.*

*Regarding the preparation of clinicians: all clinicians who participated followed a general in-company training on moral injury. While no formal standardized training protocol for the CIMI-M was in place at this stage of instrument development, all clinicians received a copy of the CIMI-M in advance and were asked to read through it carefully. Prior to each administration, the first author met individually with each clinician to walk them through the interview, explain the study procedure, and address any questions. We acknowledge that the absence of a formal training protocol is a limitation, and that future implementation of the CIMI-M would benefit from a standardized training procedure—as we discuss in the clinical implications section.*

*We have revised the manuscript as follows to make this more clear: “Recruited were (1) veterans who were on the waitlist for treatment and who, during intake, had disclosed a PMIE and/or negative moral emotions related to a traumatic event, and (2) clinicians familiar with the concept of moral injury, who were asked to administer the CIMI-M to the recruited veterans. When a veteran was already being seen by a clinician sufficiently familiar with moral injury, that clinician was invited to participate; where this was not the case, an alternative clinician with relevant expertise was approached. All clinicians who participated followed a general in-company training on moral injury. Additionally, prior to administration, all clinicians received the CIMI-M instructions manual and questions and met individually with the first author to discuss the interview and the study procedure.”*

*Regarding participant demographics: gender and nationality are reported in the manuscript. Age data were not systematically collected for this feasibility sample.*

12. Subsections for findings are very succinct, sometimes only including 1-2 sentences, at the expense of important information. Could some quotes from the table be included in text to improve the flow of the narrative?

*While this might be helpful, given the length of the manuscript (almost 10,000 words), we have chosen to not include these sentences in the main text.*

13. Often, the authors mention changes to the CIMI-M without detailing these. For example, 'Revisions included the rewording of some questions, additional follow-up questions, and a new question about self-worth.' Can you please include further information?

*We thank the reviewer for this suggestion. In response, we added an additional example of a revision made to the interview to the manuscript. This section now reads as follows: “Revisions included the rewording of some questions, additional follow-up questions, and a new question about self-worth. For example, a question about emotional coping strategies was initially followed by one follow-up question probing whether the coping behavior was helpful. Based on feedback, this was revised to include several more specific follow-up questions inquiring about coping behavior in different contexts, such as when alone or in interactions with others. Additionally, as a question about the spiritual impact of the PMIE did not resonate with non-religious veterans, we split this question into two questions: one inquiring about the impact of the PMIE on religious and spiritual beliefs and another inquiring about the loss of belief in the meaning or value of life. We also adjusted the headings and question sequencing for the interview to align with the domains of moral injury outlined by Litz et al. [5].”*

*Many of the remaining changes were relatively minor (such as the small rewording of questions for clarity or the addition of brief follow-up questions). Detailing each revision individually would add considerable length to the manuscript without substantially enhancing the reader's understanding of the development and evaluation of the CIMI-M.*

14. 'section 4: desired change and readiness for change' - This is not described earlier in the manuscript, but is interesting - explanation for this section is warranted.

*Section 4 was included for two related reasons. First, it serves to gauge the veteran's motivation and readiness to address moral injury in treatment. Engaging in treatment for moral injury can be challenging, as it requires veterans to confront deeply painful experiences and emotions that they may have been avoiding for years. Understanding a veteran's readiness for change at the outset therefore provides clinically valuable information Norman et al. (2019) [32]. Second, this section elicits the veteran's own preferences for treatment, for instance, whether they prefer individual or group treatment, whether they wish to involve family, and whether faith or spirituality is an important consideration. Together, these questions support a person-centered approach to treatment planning that incorporates the veteran's own goals, prior help-seeking efforts, and preferences for care.*

*We have added the following rationale for section 4 in the manuscript (under the section ‘interview adaptation’): “Section 4 assesses the veteran's readiness for treatment and their preferences for care (e.g. preference for individual versus group therapy or including chaplain care), to support a person-centered approach to treatment planning. Moreover, because treatment for moral injury can be challenging (requiring veterans to confront deeply painful experiences they may have been avoiding for years), understanding a veteran's readiness for change at the outset provides clinically valuable information [32].”*

15. It would be extraordinarily helpful for the interview and its instructions to be provided to interested readers to fully understand the manuscript and the development of the interview.

*The full CIMI-M, including its instructions for administration and case conceptualization template, was submitted as S2 Supporting Information.*

*We hope that you feel that our revision has satisfactorily addressed any remaining concerns and that our paper is now fit for publication.*
