# Supplementary material for: Mapping moral injury: Development and feasibility of the Clinical Interview for Moral Injury—military version (CIMI-M)
Source: PLoS One. 2026 Sep 30;21(9):e0359553. doi: 10.1371/journal.pone.0359553 (PMC13626660; doi:10.1371/journal.pone.0359553)
Supplement: Peer review history

# Peer review: Decision Letter 0

**DOI:** 10.1371/journal.pone.0359553.r001

**Authors:** Andrea Cioffi

14 Apr 2026

Dear Dr. de Goede,

Finally, the manuscript would benefit from greater clarity regarding the intended scope and application of the CIMI-M. At present, the interview is positioned as a clinical tool to support case conceptualization and treatment planning, yet it is introduced and evaluated within a research framework. A more explicit discussion of its intended use—whether primarily clinical, research-oriented, or both—would enhance the interpretability and positioning of the instrument. This is particularly important in light of the absence of diagnostic or psychometric validation at this stage.

Please submit your revised manuscript by May 29 2026 11:59PM. If you will need more time than this to complete your revisions, please reply to this message or contact the journal office at plosone@plos.org. When you're ready to submit your revision, log on to https://www.editorialmanager.com/pone/ and select the 'Submissions Needing Revision' folder to locate your manuscript file.

- A letter that responds to each point raised by the academic editor and reviewer(s). You should upload this letter as a separate file labeled 'Response to Reviewers'.
- A marked-up copy of your manuscript that highlights changes made to the original version. You should upload this as a separate file labeled 'Revised Manuscript with Track Changes'.
- An unmarked version of your revised paper without tracked changes. You should upload this as a separate file labeled 'Manuscript'.

If you would like to make changes to your financial disclosure, please include your updated statement in your cover letter. Guidelines for resubmitting your figure files are available below the reviewer comments at the end of this letter.

If applicable, we recommend that you deposit your laboratory protocols in protocols.io to enhance the reproducibility of your results. Protocols.io assigns your protocol its own identifier (DOI) so that it can be cited independently in the future. For instructions see: https://journals.plos.org/plosone/s/submission-guidelines#loc-laboratory-protocols. Additionally, PLOS ONE offers an option for publishing peer-reviewed Lab Protocol articles, which describe protocols hosted on protocols.io. Read more information on sharing protocols at https://plos.org/protocols?utm_medium=editorial-email&utm_source=authorletters&utm_campaign=protocols.

As the corresponding author, your ORCID iD is verified in the submission system and will appear in the published article. PLOS supports the use of ORCID, and we encourage all coauthors to register for an ORCID iD and use it as well. Please encourage your coauthors to verify their ORCID iD within the submission system before final acceptance, as unverified ORCID iDs will not appear in the published article. *Only*  the individual author can complete the verification step; PLOS staff *cannot*  verify ORCID iDs on behalf of authors.

We look forward to receiving your revised manuscript.

Kind regards,

Andrea Cioffi

Academic Editor

PLOS One

Journal Requirements:

When submitting your revision, we need you to address these additional requirements.

1. Please ensure that your manuscript meets PLOS ONE's style requirements, including those for file naming. The PLOS ONE style templates can be found at

https://journals.plos.org/plosone/s/file?id=wjVg/PLOSOne_formatting_sample_main_body.pdf and

https://journals.plos.org/plosone/s/file?id=ba62/PLOSOne_formatting_sample_title_authors_affiliations.pdf

2. In the online submission form, you indicated that [The data, analysis code, and research materials can be obtained upon request from the corresponding author. Due to the sensitive nature of the data, access may be limited by ethical and privacy considerations.].

All PLOS journals now require all data underlying the findings described in their manuscript to be freely available to other researchers, either 1. In a public repository, 2. Within the manuscript itself, or 3. Uploaded as supplementary information.

This policy applies to all data except where public deposition would breach compliance with the protocol approved by your research ethics board. If your data cannot be made publicly available for ethical or legal reasons (e.g., public availability would compromise patient privacy), please explain your reasons on resubmission and your exemption request will be escalated for approval.

3. Your ethics statement should only appear in the Methods section of your manuscript. If your ethics statement is written in any section besides the Methods, please move it to the Methods section and delete it from any other section. Please ensure that your ethics statement is included in your manuscript, as the ethics statement entered into the online submission form will not be published alongside your manuscript.

4. Please include captions for your Supporting Information files at the end of your manuscript, and update any in-text citations to match accordingly. Please see our Supporting Information guidelines for more information: http://journals.plos.org/plosone/s/supporting-information.

5. If the reviewer comments include a recommendation to cite specific previously published works, please review and evaluate these publications to determine whether they are relevant and should be cited. There is no requirement to cite these works unless the editor has indicated otherwise.

[Note: HTML markup is below. Please do not edit.]

Reviewers' comments:

Reviewer's Responses to Questions

**Comments to the Author**

1. Is the manuscript technically sound, and do the data support the conclusions?

Reviewer #1: Yes

Reviewer #2: Yes

**********

2. Has the statistical analysis been performed appropriately and rigorously?

Reviewer #1: Yes

Reviewer #2: I Don't Know

**********

3. Have the authors made all data underlying the findings in their manuscript fully available?

The PLOS Data policy

Reviewer #1: Yes

Reviewer #2: Yes

**********

4. Is the manuscript presented in an intelligible fashion and written in standard English?

Reviewer #1: Yes

Reviewer #2: Yes

**********

Reviewer #1: Manuscript: Mapping Moral Injury: Development and Feasibility of the Clinical Interview for Moral Injury, Military Version (CIMI-M)

Overall Assessment

This manuscript presents the development and preliminary feasibility evaluation of the Clinical Interview for Moral Injury Military Version (CIMI-M), a structured clinical interview intended to support descriptive assessment, case conceptualisation, and treatment planning for moral injury among military veterans. The study addresses an important gap in the assessment of moral injury, as existing instruments are largely self report questionnaires that may not capture the contextual, narrative, and moral meaning making dimensions central to the construct.

The manuscript makes a valuable contribution by proposing a clinician administered interview format grounded in multiple theoretical models of moral injury and evaluated through a feasibility study involving clinicians and veterans. The development process is iterative and stakeholder informed, and the authors provide transparency regarding the conceptual and methodological decisions involved.

Overall, the manuscript represents a useful early step toward structured clinical assessment of moral injury. However, several conceptual, methodological, and reporting issues should be addressed to strengthen the manuscript and clarify the clinical and scientific implications of the proposed instrument.

Major Comments

1. Conceptual Clarification of Moral Injury

The manuscript appropriately acknowledges that moral injury remains a contested construct, with ongoing debates about its boundaries and differentiation from related disorders such as PTSD and depression although these are rapidly resolving. However, this conceptual uncertainty raises an important question that is not fully addressed in the paper: what exactly the CIMI-M is intended to measure.

The interview is presented as a descriptive tool rather than a diagnostic instrument, yet it is also positioned as assessing “moral injury.” Because they argue the field lacks consensus on diagnostic boundaries, the authors should clarify whether the interview primarily assesses:

• exposure to potentially morally injurious experiences (PMIEs),

• the psychological and existential impact of such experiences, or

• a broader moral injury syndrome.

Greater clarity about this distinction would help readers understand how the CIMI-M fits within existing moral injury measurement frameworks.

2. Distinction Between Moral Injury and Trauma Related Disorders

The authors note the ongoing debate regarding whether moral injury is distinct from PTSD and depression, which I think is resolved, while it can be comorbid, it is the same disorder neurologically, MI is active in the DMN whereas PTSD is active in the SMN, symptom differences are also reflected in the same way. However, if the authors want to make the case of debate and uncertainty then please clarify. Without this clarification, there is a risk that the CIMI-M may function primarily as a trauma narrative exploration tool rather than a distinct moral injury assessment instrument.

3. Sample Size and Feasibility Study Design

The feasibility study included eight veterans and seven clinicians, which is acceptable for exploratory feasibility testing but limits the interpretability of findings. The authors appropriately describe the study as preliminary; however, several aspects of the design could be clarified:

• The sampling strategy appears to involve treatment seeking veterans already identified as having PMIEs, which may bias the evaluation toward positive perceptions of the interview.

• The clinicians involved appear to have prior familiarity with moral injury, which may further bias feasibility ratings.

• The use of mean evaluation scores (e.g., 7.63 for veterans and 8.41 for clinicians) is reported descriptively but not contextualised with benchmarks or comparison measures.

It would strengthen the manuscript to emphasise that the results represent acceptability among a highly selected population rather than general clinical feasibility.

4. Evaluation Measures

The study employs the Debriefing Instrument for Patients (DIP) and Debriefing Instrument for Clinicians (DIC), originally developed for evaluation of the Cultural Formulation Interview (CFI). While the adaptation of these instruments is reasonable, the manuscript should provide more discussion of:

• their validity in this context,

• the implications of removing negatively worded items, and

• whether the constructs measured (feasibility, acceptability, clinical utility) fully capture the interview performance.

Additionally, since the CIMI-M is designed to inform treatment planning, it would be useful to propose as future research an outcome based validation, such as whether its use improves case formulation or treatment matching.

5. Clinical Implementation Considerations

The authors appropriately note that implementation of descriptive interviews may be challenging in clinical systems that prioritise symptom based diagnostics.

However, the manuscript could benefit from comments around the practical issues related to implementation, including:

• training requirements for clinicians unfamiliar with moral injury

• integration with existing trauma assessments

• time burden within standard intake processes

• cross cultural adaptation for non-military populations.

These issues are particularly relevant if the authors intend the CIMI-M to be widely adopted in clinical settings.

Minor Comments

1. Terminology Consistency

The manuscript alternates between referring to the interview as assessing PMIE exposure, moral injury, and impact of moral injury. Greater terminological consistency would improve conceptual clarity.

2. Spiritual Domain

Both veterans and clinicians reportedly found questions regarding the spiritual impact of PMIEs less relevant in some cases. The authors might consider discussing whether the spiritual domain should be framed more broadly (e.g., meaning, values, or worldview disruption) to increase cultural applicability.

3. Interview Length

The reported duration of approximately 90 minutes may present a barrier in some clinical environments. The manuscript could discuss whether a short form version might be developed.

4. International Applicability

The sample consisted entirely of Dutch veterans, which may limit generalizability.

Future research should consider testing the instrument across different military cultures and healthcare systems.

Strengths of the Manuscript

Despite the limitations noted above, the manuscript has several important strengths:

1. Addresses a clear gap in the field

There is currently no structured clinical interview designed specifically for moral injury assessment.

2. Strong theoretical grounding

The interview integrates multiple moral injury models, including the frameworks proposed by Litz et al. (2009), Farnsworth et al. (2017), and Litz et al. (2022).

3. Stakeholder informed development

The iterative design process included clinicians, researchers, and veterans.

4. Clinically meaningful focus

The emphasis on narrative meaning making and case conceptualisation aligns well with emerging therapeutic approaches to moral injury.

Recommendations

The manuscript would benefit from the following revisions:

1. Clarify the conceptual distinction between PMIE exposure, moral distress, and moral injury within the interview.

2. Expand discussion of how clinicians should differentiate moral injury from PTSD or depression during assessment.

3. Emphasise the preliminary nature of the feasibility findings.

4. Provide more detail regarding implementation and training considerations.

5. Clarify the intended clinical role of the CIMI-M within existing assessment frameworks.

Reviewer #2: Important topic

Innovative product

Explicit layout of a development process of a descriptive/clinical interview. Other projects/other topics could follow this model for high impact but hard to define and predict conditions that impact daily life and clinical care.

Not sure there is exactly proof of concept but certain worth more more work--this is not to say this work does not show a rigorous, well-thought out project/pilot.

More female participants seems a critical future direction

Minor comment

Haynes et al. (1995) is not the references

**********

what does this mean?). If published, this will include your full peer review and any attached files.

If you choose “no”, your identity will remain anonymous but your review may still be made public.

**Do you want your identity to be public for this peer review?**  For information about this choice, including consent withdrawal, please see our Privacy Policy

Reviewer #1: No

Reviewer #2: No

**********

[NOTE: If reviewer comments were submitted as an attachment file, they will be attached to this email and accessible via the submission site. Please log into your account, locate the manuscript record, and check for the action link "View Attachments". If this link does not appear, there are no attachment files.]

To ensure your figures meet our technical requirements, please review our figure guidelines: https://journals.plos.org/plosone/s/figures

You may also use PLOS’s free figure tool, NAAS, to help you prepare publication quality figures: https://journals.plos.org/plosone/s/figures#loc-tools-for-figure-preparation.

NAAS will assess whether your figures meet our technical requirements by comparing each figure against our figure specifications.

# Author comment: Author response to Decision Letter 1

**DOI:** 10.1371/journal.pone.0359553.r002

8 Jul 2026

Dear Editor and reviewers,

Thank you for the opportunity to revise and resubmit our manuscript ‘Mapping Moral Injury: Development and Feasibility of the Clinical Interview for Moral Injury—Military Version (CIMI-M)’. We are grateful for the careful reviews and have adapted the manuscript according to your suggestions, marking changes in the revised document. Please see our response to your comments below.

Academic Editor:

1. A central issue concerns the inherently preliminary nature of the empirical evaluation. The feasibility study is based on a very small and highly specific sample, comprising eight veterans and seven clinicians recruited from two specialized centers. While such a sample size may be acceptable for an initial feasibility assessment, it substantially limits the generalizability of the findings and precludes any robust conclusions regarding the broader applicability of the instrument. Although this limitation is acknowledged in the manuscript, the discussion and conclusions at times appear more affirmative than warranted by the data. I would therefore encourage you to more explicitly frame the findings as exploratory and preliminary, and to ensure that all claims regarding feasibility, acceptability, and clinical utility are carefully calibrated to the limited evidentiary base. Relatedly, the evaluation of the CIMI-M focuses exclusively on perceived feasibility, acceptability, and clinical utility, without any formal assessment of psychometric properties. While this is consistent with a feasibility study design, the absence of data on reliability, validity, and structural characteristics should be more clearly emphasized as a major limitation. In particular, it would be important to clarify that the instrument, at this stage, should not be interpreted as a validated clinical or research tool, but rather as a promising prototype requiring further empirical testing.

Thank you for these suggestions. In response to your comment regarding the preliminary findings of this study, we have revised the manuscript in several places:

• In the abstract: “We found preliminary evidence that the CIMI-M is a feasible, acceptable, and clinically useful descriptive interview.”

• In the introduction: “We also provide the results of an exploratory study of the CIMI-M, which entailed an examination of veteran and therapist’ perspectives of its feasibility, acceptability, and clinical utility, and which was used to modify the interview.”

• In the discussion: “The selective sample of clinicians and veterans who participated in this preliminary evaluation deemed the CIMI-M feasible, acceptable, and clinically useful.”

• In the conclusion: “In a preliminary feasibility study, the CIMI-M was positively evaluated by veterans and clinicians and subsequently refined based on their feedback.”

As for your observation regarding the lack of formal assessment of the interview’s psychometric properties: Because the CIMI-M is a semi-structured clinical interview designed to support moral injury-related case formulation and treatment planning, rather than a diagnostic instrument or continuous symptom scale, conventional psychometric indices such as internal consistency, factor structure, and cut-score validation are not the most appropriate initial evidentiary standards. We have revised the manuscript to clarify the instrument’s intended use and to distinguish feasibility, acceptability, content validity, and clinical utility from formal psychometric validation. We also now identify interrater reliability of structured formulation outputs as an important next step.

In a future study, we intend to examine interrater reliability. Our plan is to have different trained raters independently review the same recorded CIMI-M interviews and complete the reporting template. Agreement could be evaluated for categorical determinations such as PMIE presence/absence, PMIE type, and presence/absence of clinically salient impact within each domain using percent agreement. We would employ a weighted kappa to determine agreement relative to chance. We could also have raters assign dimensional ratings of domain severity or clinical significance and examine agreement using intraclass correlation coefficients.

In the discussion, we have added language about subsequent studies that will be examining interrater reliability of the structured formulation outputs and, eventually, predictive or treatment-planning utility. We have also revised the discussion to clarify that the CIMI-M should be regarded as a promising prototype. We have revised the text as follows:

“Although this study showed promising preliminary results, the CIMI-M should be regarded as a prototype at this stage of development. The CIMI-M is not a continuous symptom severity (or frequency) scale, consequently, conventional psychometric indices such as internal consistency reliability and construct validity are not germane. However, future research should examine interrater reliability, cross-cultural content validity, and criterion-related validity with respect to concurrent and predictive validity. For example, trained raters could independently review the same recorded CIMI-M interviews and complete the reporting template. Agreement could be evaluated for categorical determinations such as PMIE presence/absence, PMIE type, and presence/absence of clinically salient impact within each domain using a weighted kappa to determine agreement relative to chance. With respect to concurrent validity, the CIMI-M could be administered to various groups and the caseness variable could be compared with continuous scores on a moral injury outcome scale. An odds ratio per unit increase in scale score could index the strength of association the predicted probabilities of caseness across score ranges could be determined.”

2. A further concern pertains to the potential for selection and desirability biases. Participants were recruited within specialized clinical settings and included clinicians already familiar with, or interested in, the concept of moral injury. As you note, this may have led to more favorable evaluations of the interview. However, this issue would benefit from a more critical and integrated discussion, particularly in relation to how such biases may have influenced both quantitative ratings and qualitative feedback. Expanding this reflection would strengthen the credibility and transparency of the manuscript.

In response to your comment, as well as comment 3 by reviewer 1 below, we have revised our discussion section as follows: "Lastly, veterans identified as having experienced a PMIE and highly motivated clinicians with an interest in moral injury participated in the study. This may have introduced a positive bias, potentially leading to more favorable ratings of the CIMI-M. However, at this stage of interview development, administering the full interview required veterans with such experiences, while asking clinicians less familiar with moral injury to administer the interview would have risked conflating unfamiliarity with the construct with limitations of the instrument itself. The face-to-face debrief interview may also have introduced social desirability bias, potentially leading to more positive evaluations than participants might have provided in an anonymous format. Taken together, these findings reflect a preliminary evaluation of acceptability and feasibility within a highly selected, clinically relevant population and do not provide evidence of general feasibility across diverse settings and practitioners. For future evaluations of the interview, it would be valuable to include clinicians less familiar with moral injury, as well as veterans from more diverse backgrounds.”

3. Finally, the manuscript would benefit from greater clarity regarding the intended scope and application of the CIMI-M. At present, the interview is positioned as a clinical tool to support case conceptualization and treatment planning, yet it is introduced and evaluated within a research framework. A more explicit discussion of its intended use—whether primarily clinical, research-oriented, or both—would enhance the interpretability and positioning of the instrument. This is particularly important in light of the absence of diagnostic or psychometric validation at this stage.

We thank the editor for this comment. The CIMI-M is positioned throughout the manuscript as a clinical tool designed to support case conceptualization and treatment planning. Although research has identified severity score thresholds on moral injury outcome questionnaires to indicate caseness, moral injury does not currently have a configural diagnostic decision rule nor is it codified as a mental disease. However, the purpose of the CIMI-M is to guide case formulation and treatment planning, which would be valuable even if there was a clinical interview analogous to the CAPS-5 that determined the diagnosis of moral injury. At this point in the clinical science of moral injury, if clinicians want to identify probable clinical caseness as a gatekeeping function for initiating a CIMI-M, they can use the Moral Injury Outcome Scale (MIOS), which for veterans has a caseness threshold of >31.

Clinical contexts can equally be research contexts (e.g., quality improvement, pragmatic point of care effectiveness studies, etc.). We did a research study of the CIMI-M in a clinical context in order to explore the interview's feasibility, acceptability, and clinical utility. Participants were real patients to whom the interview was administered following intake and prior to the start of treatment. When moral injury treatment was indicated, patients received it. We used the term research because this was a formal evaluation in a clinical context, not to repurpose the CIMI-M as a research instrument.

Having said the above, we believe that the CIMI-M could be used as a research tool. For example, when a configural decision rule is generated for diagnosis, the CIMI-M could be readily adapted as a clinical interview because the domains of impact constitute symptom classes (e.g., the presence of moral emotions). If that happened, the CIMI-M could be used to identify cases based on a diagnostic rule for research purposes.

When submitting your revision, we need you to address these additional requirements.

1. Please ensure that your manuscript meets PLOS ONE's style requirements, including those for file naming. The PLOS ONE style templates can be found at

https://journals.plos.org/plosone/s/file?id=wjVg/PLOSOne_formatting_sample_main_body.pdf and

https://journals.plos.org/plosone/s/file?id=ba62/PLOSOne_formatting_sample_title_authors_affiliations.pdf

We have changed our manuscript according to the PLOS ONE’s style requirements.

2. In the online submission form, you indicated that [The data, analysis code, and research materials can be obtained upon request from the corresponding author. Due to the sensitive nature of the data, access may be limited by ethical and privacy considerations.]. All PLOS journals now require all data underlying the findings described in their manuscript to be freely available to other researchers, either 1. In a public repository, 2. Within the manuscript itself, or 3. Uploaded as supplementary information. This policy applies to all data except where public deposition would breach compliance with the protocol approved by your research ethics board. If your data cannot be made publicly available for ethical or legal reasons (e.g., public availability would compromise patient privacy), please explain your reasons on resubmission and your exemption request will be escalated for approval.

Thank you for raising this. We would like to request an exemption from the public data availability requirement. The data underlying this study consist of clinical interview recordings and transcripts obtained from a clinical veteran population. Participants provided informed consent under the explicit condition that their data would remain confidential and would not be made publicly available. Public deposition of these data would therefore directly breach the terms of consent and compromise participant privacy. Furthermore, public availability of the data is not permitted under the protocol approved by our research ethics board. Sharing potentially re-identifiable clinical interview data in a public repository would violate both our ethical approval conditions and Dutch privacy legislation. For these reasons, we maintain that data can only be made available upon reasonable request to the corresponding author, subject to ethical and privacy review. We trust this explanation provides sufficient grounds for an exemption and are happy to provide further documentation if required.

3. Your ethics statement should only appear in the Methods section of your manuscript. If your ethics statement is written in any section besides the Methods, please move it to the Methods section and delete it from any other section. Please ensure that your ethics statement is included in your manuscript, as the ethics statement entered into the online submission form will not be published alongside your manuscript.

Our manuscript originally contained a section called ‘Feasibility Study’ following the section ‘Development of the CIMI-M’. We have renamed this section ‘Methods’. The ethics statement is within this section.

4. Please include captions for your Supporting Information files at the end of your manuscript, and update any in-text citations to match accordingly. Please see our Supporting Information guidelines for more information: http://journals.plos.org/plosone/s/supporting-information.

We have revised this as requested.

Reviewer #1:

Overall Assessment

This manuscript presents the development and preliminary feasibility evaluation of the Clinical Interview for Moral Injury Military Version (CIMI-M), a structured clinical interview intended to support descriptive assessment, case conceptualisation, and treatment planning for moral injury among military veterans. The study addresses an important gap in the assessment of moral injury, as existing instruments are largely self-report questionnaires that may not capture the contextual, narrative, and moral meaning making dimensions central to the construct.

The manuscript makes a valuable contribution by proposing a clinician administered interview format grounded in multiple theoretical models of moral injury and evaluated through a feasibility study involving clinicians and veterans. The development process is iterative and stakeholder informed, and the authors provide transparency regarding the conceptual and methodological decisions involved.

Overall, the manuscript represents a useful early step toward structured clinical assessment of moral injury. However, several conceptual, methodological, and reporting issues should be addressed to strengthen the manuscript and clarify the clinical and scientific implications of the proposed instrument.

Thank you for your positive evaluation of our manuscript.

Major Comments

1. Conceptual Clarification of Moral Injury

The manuscript appropriately acknowledges that moral injury remains a contested construct, with ongoing debates about its boundaries and differentiation from related disorders such as PTSD and depression although these are rapidly resolving. However, this conceptual uncertainty raises an important question that is not fully addressed in the paper: what exactly the CIMI-M is intended to measure.

The interview is presented as a descriptive tool rather than a diagnostic instrument, yet it is also positioned as assessing “moral injury.” Because they argue the field lacks consensus on diagnostic boundaries, the authors should clarify whether the interview primarily assesses:

• exposure to potentially morally injurious experiences (PMIEs),

• the psychological and existential impact of such experiences, or

• a broader moral injury syndrome.

Greater clarity about this distinction would help readers understand how the CIMI-M fits within existing moral injury measurement frameworks.

We thank the reviewer for this constructive comment. To clarify, the CIMI-M includes all three elements the reviewer distinguishes, but in a hierarchical manner: PMIE exposure as precipitant, the impact of that experience across the six domains of impact as identified by Litz et al. (2022) as constituting the syndrome of moral injury as an outcome. Moral injury is like PTSD in that exposure is a gatekeeper to assessing outcome (symptom) presence and severity. At the same time, because there is no consensus about a configural set of criteria for a moral injury diagnosis, the CIMI-M is not designed to identify moral injury cases formally. It is not a diagnostic tool. Instead, the interview manual provides structured guidance for determining probable caseness based on a clinical judgment about whether the interviewee likely experiences clinically significant moral injury that warrants treatment. This judgment is based on four questions: (1) Has the interviewee experienced a high-stakes and currently distressing PMIE? (2) Is the PMIE associated with current functionally interfering problems within more than one domain of impact? (3) Are these problems new onset (i.e. began or worsened following the PMIE?) (4) Is there evidence that recovery has been impeded such that moral injury constitutes a pressing clinical problem?

The CIMI-M thus occupies a middle ground: it does not generate a formal diagnosis, but it does provide a structured basis for clinical judgment of whether an individual endorses clinically significant moral injury symptoms requiring treatment. To clarify this, we have rewritten the sentence in the introduction outlining our aims: “Our objective was to create a clinical interview for moral injury that: (1) assesses exposure to different PMIE types as well as the impact of such experiences across the six moral injury domains identified by Litz et al. ([5]; (2) allows for a descriptive dialogue that includes the patient’s own perspective; and (3) facilitates case conceptualization, treatment planning, and an exploratory configural caseness determination.”

We also also added the following to the section ‘instrument aims’: “We experimentally defined clinical caseness as: (1) endorsement of at least one high-stakes and currently distressing PMIE; (2) reports of functionally interfering problems in at least two domains of moral injury; (3) that are new onset; and (4) for which recovery has been impeded or is unlikely without clinical intervention. This configural decision rule for caseness is by definition not a formal diagnosis of a mental disease (moral injury is not currently in a disease taxonomy), but it formalizes the assessment of whether an individual warrants treatment for a potential moral injury within the context of data collected in the CIMI-M.”

2. Distinction Between Moral Injury and Trauma Related Disorders

The authors note the ongoing debate regarding whether moral injury is distinct from PTSD and depression, which I think is resolved, while it can be comorbid, it is the same disorder neurologically, MI is active in the DMN whereas PTSD is active in the SMN, symptom differences are also reflected in the same way. However, if the authors want to make the case of debate and uncertainty then please clarify. Without this clarification, there is a risk that the CIMI-M may function primarily as a trauma narrative exploration tool rather than a distinct moral injury assessment instrument.

We assume this comment intended to say “not the same disorder neurologically”, which aligns with the argument that follows.

We agree that there is growing evidence supporting the distinctness of moral injury from PTSD and depression. However, we respectfully maintain that characterizing this debate as fully resolved overstates the current consensus. We appreciate the reviewer's concern that without sufficient differentiation, the CIMI-M risks functioning as a general trauma narrative tool rather than a distinct moral injury instrument. We believe the CIMI-M's distinctiveness as a moral injury instrument rests on three features: its explicit focus on violated moral beliefs and values as the defining characteristic of PMIEs; its structured assessment of the six moral injury domains identified by Litz et al. (2022); and its caseness determination approach (as explained in response to comment 1), which is grounded in functional impairment across multiple moral injury-specific domains rather than trauma symptoms more broadly.

To emphasize this, we have added a brief clarification in the discussion noting that, “By clearly identifying the presence of a PMIE, assessing moral values that were violated, and determining to what extent current problems across moral injury domains are attributable to these PMIEs also helps distinguish these problems from PTSD and depression.”

3. Sample Size and Feasibility Study Design

The feasibility study included eight veterans and seven clinicians, which is acceptable for exploratory feasibility testing but limits the interpretability of findings. The authors appropriately describe the study as preliminary; however, several aspects of the design could be clarified:

• The sampling strategy appears to involve treatment seeking veterans already identified as having PMIEs, which may bias the evaluation toward positive perceptions of the interview.

• The clinicians involved appear to have prior familiarity with moral injury, which may further bias feasibility ratings.

• The use of mean evaluation scores (e.g., 7.63 for veterans and 8.41 for clinicians) is reported descriptively but not contextualised with benchmarks or comparison measures.

It would strengthen the manuscript to emphasise that the results represent acceptability among a highly selected population rather than general clinical feasibility.

We thank the reviewer for these thoughtful observations. We address each point in turn:

• We acknowledge that recruiting treatment-seeking veterans already identified as having PMIEs may bias evaluations towards more positive perceptions of the interview. However, it is important to note that this sampling strategy was not only pragmatic but deemed necessary: the CIMI-M is designed to be administered to individuals who have experienced a PMIE and administering it to veterans without such experiences would have precluded evaluation of the full interview. At this stage of development, testing the instrument with its intended population was therefore the appropriate methodological choice.

• We agree that clinicians with prior familiarity with moral injury may have rated the interview more favorably than clinicians without such background. At the same time, this was a deliberate choice: at this early stage of instrument development, clinicians with moral injury expertise were best positioned to evaluate the interview's content validity and provide constructive feedback on its clinical utility. Involving clinicians less familiar with moral injury at this stage would have risked conflating unfamiliarity with the construct with limitations of the instrument itself. We now acknowledge in the limitations section that future evaluations should include clinicians with varying levels of familiarity with moral injury to assess its broader usability.

• We understand the reviewer's point that mean scores require contextualization to be interpretable. However, given the novelty of this instrument, there are no established benchmarks or directly comparable feasibility studies against which to contextualize these scores. The mean scores were therefore intended to provide a general impression of overall evaluation rather than a precise psychometric estimate and should be interpreted alongside the qualitative findings.

We have addressed these concerns by rewriting our limitations section as follows: "Lastly, veterans identified as having experienced a PMIE and highly motivated clinicians with an interest in moral injury participated in the study. This may have introduced a positive bias, potentially leading to more favorable ratings of the CIMI-M. However, at this stage of interview development, administering the full interview required veterans with such experiences, while asking clinicians less familiar with moral injury to administer the interview would have risked conflating unfamiliarity with the construct with limitations of the instrument itself. The face-to-face debrief interview may also have introduced social desirability bias, potentially leading to more positive evaluations than participants might have provided in an anonymous format. Taken together, these findings are best understood as reflecting a preliminary evaluation of acceptability and feasibility within a highly selected, clinically relevant population, rather than as evidence of general clinical feasibility across diverse settings and practitioners. For future evaluations of the interview, it would be valuable to include clinicians less familiar with moral injury, as well as veterans from more diverse backgrounds.”

4. Evaluation Measures

The study employs the Debriefing Instrument for Patients (DIP) and Debriefing Instrument for Clinicians (DIC), originally developed for evaluation of the Cultural Formulation Interview (CFI). While the adaptation of these instruments is reasonable, the manuscript should provide more discussion of:

• their validity in this context,

• the implications of removing negatively worded items, and

• whether the constructs measured (feasibility, acceptability, clinical utility) fully capture the interview performance.

Additionally, since the CIMI-M is designed to inform treatment planning, it would be useful to propose as future research an outcome based validation, such as whether its use improves case formulation or treatment matching.

We thank the reviewer for these observations. We address each point in turn.

• We acknowledge that the DIP and DIC were originally developed and validated for evaluation of the CFI, and that their use in the context of the CIMI-M therefore requires some justification. We chose these instruments because the CIMI-M was explicitly modeled in part on the CFI as a descriptive, non-diagnostic clinical interview, making the DIP and DIC the most conceptually appropriate available measures for this purpose. There are, to our knowledge, no other instruments for evaluating the feasibility, acceptability, and clinical utility of clinical interviews of this kind, and developing a new instrument was beyond the scope of this study. The DIP and DIC also demonstrated generally good psychometric properties in the CFI field trial (Lewis-Fernandez et al., 2017). Although their validity has not been examined specifically in the context of moral injury assessment, we believe the conceptual similarities between the CFI and the CIMI-M as interviews support the use of DIP and DIC in the current context. Nevertheless, we acknowledge that the psychometric properties of the DIP and DIC as used here (including the adapted Dutch translation and the removal of two negatively worded items) have not been independently established, and findings should be interpreted with this in mind.

• The two negatively worded DIP items were removed because prior research demonstrated that they negatively impacted internal consistency, and their removal was explicitly recommended by the scale's developer (Lewis-Fernandez et al., 2017). We acknowledge that removing these items means feasibility and acceptability were each assessed with only two items. However, given the small sample size and the preliminary nature of this study, the quantitative scores were never intended as precise psychometric estimates. Rather, they served as a broad indicator of overall evaluation, complementing the qualitative interview data which formed the primary basis for our conclusions.

• We believed feasibility, acceptability, and clinical utility to be among the most relevant constructs for this early stage of instrument evaluation. However, we agree that interview performance could also have been captured by constructs such as inter-interviewer reliability or convergent validity.

We have added a brief acknowledgment of these concerns in the limitations section: “Another limitation concerns the use of the DIP and DIC, which were developed for the CFI and whose psychometric properties have not been established for use with other instruments. However, given the preliminary nature and small sample size of this study, the quantitative scores should be interpreted as broad indicators of the overall evaluation and were intended to complement the qualitative findings. Future evaluations of the CIMI-M would benefit from more comprehensive psychometric assessment, including inter-interviewer reliability and content validity.”

As for your suggestion for future research, we have revised a sentence in our limitation section as follows: “More firmly establishing the clinical utility of the CIMI-M would also necessitate long-term data collection, ideally extending post-therapy, to assess if and how the interview improves case formulation, treatment matching, or clinical outcomes.”

5. Clinical Implementation Considerations

The authors appropriately note that implementation of descriptive interviews may be challenging in clinical systems that prioritize symptom based diagnostics.

However, the manuscript could benefit from comments around the practical issues related to implementation, including:

• training requirements for clinicians unfamiliar with moral injury

• integration with existing trauma assessments

• time burden within standard intake processes

• cross cultural adaptation for non-military populations.

These issues are particularly relevant if the authors intend the CIMI-M to be widely adopted in clinical settings.

In response to your comment, we added the following paragraphs to the clinical implications section: “The CIMI-M was designed to complement existing intake procedures and should only be administered if there is an indication a veteran experienced a PMIE, for example at intake. We recommend administering the MIOS [5] prior to the CIMI-M to screen for PMIE exposure, to determine whether the interview is warranted. The CIMI-M may also be used alongside PTSD assessments such as the CAPS-5 when assessment of both PTSD and moral injury is deemed clinically relevant. at least 90 minutes, clinicians should. Additionally, the CIMI-M's explicit focus on violated moral values, PMIE attribution, and moral emotions may help clinicians distinguish morally injurious responses from PTSD and depression. Given the interview duration of carefully consider participant burden when combining multiple assessments. Importantly, however, clinicians anecdotally reported after the study that administering the CIMI-M prior to treatment saved time during treatment, as the CIMI-M already clarified violated moral beliefs and expectations, deepened veterans' insight into their difficulties, and facilitated more targeted intervention selection.”

And: “We particularly recommend training for clinicians with limited familiarity with moral injury. For clinicians in the Netherlands, we developed two training videos in collaboration with the Netherlands Veterans Institute (each approximately 30 minutes), available online, covering the concept of moral injury and the administration of the interview, respectively. In-person training, as is common practice for the CAPS-5, may provide additional value by allowing clinicians to address questions around administration.

Finally, while the current version was developed for military veterans, adaptation for non-military populations would be valuable. This would require modification of the context questions and, possibly, the domains of impact—as the study that established the moral injury domains relied on data from military populations exclusively [5].”

Minor Comments

A. Terminology Consistency

The manuscript alternates between referring to the interview as assessing PMIE exposure, moral injury, and impact of moral injury. Greater terminological consistency would improve conceptual clarity.

The interview assesses both PMIEs and their impact, defined as moral injury. In response to your comment, we have revised several sentences in the manuscript to clarify this, e.g.: “There is currently no clinical interview to assess exposure to potentially morally injurious experiences (PMIEs) and moral injury.”

And: “The CIMI-M facilitates the formulation of a comprehensive case conceptualization that encompasses all key elements relevant to moral injury, including a detailed description of the PMIE, an overview of the violated moral values, and an assessment of the impact of the PMIE across domains of moral injury, including coping strategies for dealing with moral pain.”

B. Spiritual Domain

Both veterans and clinicians reportedly found questions regarding the spiritual impact of PMIEs less relevant in some cases. The authors might consider discussing whether the spiritual domain should be framed more broadly (e.g., meaning, values, or worldview disruption) to increase cultural applicability.

We thank the reviewer for this suggestion. As noted in our manuscript, some veterans and clinicians indeed found the question about a changed relationship with religion or spirituality less relevant. For this reason, we added a question addressing loss of belief in the meaning or value of life to capture existential impact in a way that resonates with secular or non-religious respondents. We would also like to clarify that the "spiritual domain" in the Litz et al. (2022) moral injury framework is already framed more broadly than religion per se: it encompasses "alterations in individuals' beliefs about life meaning or purpose” including “loss of belief that life is precious” (Litz et al., 2022, supplement). The CIMI-M's operationalization of this domain reflects that broader framing.

C. Interview Length

The reported duration of approximately 90 minutes may present a barrier in some clinical environments. The manuscript could discuss whether a short form version might be developed.

We thank the reviewer for this observation. We acknowledge that a 90-minute interview may present a practical barrier in some clinical settings. However, one central aim of the interview is to provide veterans with dedicated time to share their experiences in depth (a quality that was appreciated by veterans in our feasibility study, who shared that the CIMI-M helped create the supportive environment they needed for sharing their story). Reducing the interview to a shorter format risks undermining precisely this quality. As noted in response to comment 5, clinicians also shared after the study that administering the CIMI-M helped them save time during treatment.

To address the concern that 90 minutes may present a barrier in some clinical environments, we have added the following sentence to the discussion section of our manuscript: “Moreover, while a minimum duration of 90 minutes may be considered lengthy in time-pressured clinical settings, providing veterans with sufficient time to share their experiences in depth was a central aim of the CIMI-M that was explicitly appreciated by veterans.”

D. International Applicability

The sample consisted entirely of Dutch veterans, which may limit generalizability.

Future research should consider testing the instrument across different military cultures and healthcare systems.

In response to your comment, we have revised one sentence in our discussion as follows: “Therefore, to determine whether the acceptability, feasibility, and clinical utility of the CIMI-M can be generalized across various settings and populations, more systematic research is required, preferably across different countries, military cultures, and healthcare systems, with participants of different genders and backgrounds.”

Strengths of the Manuscript

Despite the limitations noted above, the manuscript has several important strengths:

1. Addresses a clear gap in the field

There is currently no structured clinical interview designed specifically for moral injury assessment.

2. Strong theoretical grounding

The interview integrates multiple moral injury models, including the frameworks proposed by Litz et al. (2009), Farnsworth et al. (2017), and Litz et al. (2022).

3. Stakeholder informed development

The iterative design process included clinicians, researchers, and veterans.

4. Clinically meaningful focus

The emphasis on narrative meaning making and case conceptualisation aligns well with emerging therapeutic approaches to moral injury.

Thank you for pointing out these strengths in our manuscript.

Recommendations

The manuscript would benefit from the following revisions:

1. Clarify the conceptual distinction between PMIE exposure, moral distress, and moral injury within the interview.

We have made changes to our manuscript as described in response to minor comment A.

2. Expand discussion of how clinicians should differentiate moral injury from PTSD or depression during assessment.

We thank the reviewer for this suggestion. We have addressed this concern in two places in the manuscript. We have made some adjustments in the manuscript to stress that the interview aims to link the assessed impact to the experience of a PMIE and moral values that were violated, please see our response to comment 2. We have also added discussion of how the interview can be integrated with existing trauma assessments, please see our response to comment 5.

3. Emphasize the preliminary nature of the feasibility findings.

Please see our response to comment 1 from the academic editor.

4. Provide more detail regarding implementation and training considerations.

In response to your comment, we have provided more detail regarding this (please see our response to comment 5).

5. Clarify the intended clinical role of the CIMI-M within existing assessment frameworks.

The CIMI-M is intended to be used following intake (and/or initial screening with self-report instruments such as the MIOS), as a comprehensive second-stage assessment that informs case conceptualization and treatment planning. It may be combined with other diagnostic interviews where differentiation between moral injury and classifications such as PTSD is indicated, although differential diagnosis is not the aim of this interview (as also described in response to comment 5 and minor comment B).

Reviewer #2:

Explicit layout of a development process of a descriptive/clinical interview. Other projects/other topics could follow this model for high impact but hard to define and predict conditions that impact daily life and clinical care.

Not sure there is exactly proof of concept but certain worth more more work--this is not to say this work does not show a rigorous, well-thought out project/pilot.

More female participants seems a critical future direction

We thank the reviewer for this comment. We have revised the manuscript throughout to more explicitly emphasize the preliminary nature of the feasibility findings, as also noted in response to the comments by the editor and other reviewer. We refer to the importance of including more female participants in future studies with the CIMI-M in the discussion section of our manuscript: “Therefore, to determine whether the acceptability, feasibility, and clinical utility of the CIMI-M can be generalized across various settings and populations, more systematic research is required, preferably across different countries, military cultures, and healthcare systems, with participants of different genders and backgrounds.”

Minor comment

Haynes et al. (1995) is not the references

We thank the reviewer for pointing this out, we have added this reference.

Attachment Submitted filename: Response to Reviewers.docx

# Peer review: Decision Letter 1

**DOI:** 10.1371/journal.pone.0359553.r003

**Authors:** Andrea Cioffi

7 Aug 2026

Dear Dr. de Goede,

Thank you for submitting your manuscript to PLOS ONE. After careful consideration, we feel that it has merit but does not fully meet PLOS ONE’s publication criteria as it currently stands. Therefore, we invite you to submit a revised version of the manuscript that addresses the points raised during the review process.

We note that some of the comments of the reviewers refer to specific articles for you to cite. Please note that it is not mandatory that you cite these specific articles and you are welcome to seek alternatives manuscripts in the literature that are relevant to your manuscript’s content.

Please submit your revised manuscript by Sep 21 2026 11:59PM. If you will need more time than this to complete your revisions, please reply to this message or contact the journal office at plosone@plos.org. When you're ready to submit your revision, log on to https://www.editorialmanager.com/pone/ and select the 'Submissions Needing Revision' folder to locate your manuscript file.

- A letter that responds to each point raised by the academic editor and reviewer(s). You should upload this letter as a separate file labeled 'Response to Reviewers'.
- A marked-up copy of your manuscript that highlights changes made to the original version. You should upload this as a separate file labeled 'Revised Manuscript with Track Changes'.
- An unmarked version of your revised paper without tracked changes. You should upload this as a separate file labeled 'Manuscript'.

If you would like to make changes to your financial disclosure, please include your updated statement in your cover letter. Guidelines for resubmitting your figure files are available below the reviewer comments at the end of this letter.

If applicable, we recommend that you deposit your laboratory protocols in protocols.io to enhance the reproducibility of your results. Protocols.io assigns your protocol its own identifier (DOI) so that it can be cited independently in the future. For instructions see: https://journals.plos.org/plosone/s/submission-guidelines#loc-laboratory-protocols. Additionally, PLOS ONE offers an option for publishing peer-reviewed Lab Protocol articles, which describe protocols hosted on protocols.io. Read more information on sharing protocols at https://plos.org/protocols?utm_medium=editorial-email&utm_source=authorletters&utm_campaign=protocols.

As the corresponding author, your ORCID iD is verified in the submission system and will appear in the published article. PLOS supports the use of ORCID, and we encourage all coauthors to register for an ORCID iD and use it as well. Please encourage your coauthors to verify their ORCID iD within the submission system before final acceptance, as unverified ORCID iDs will not appear in the published article. *Only*  the individual author can complete the verification step; PLOS staff *cannot*  verify ORCID iDs on behalf of authors.

We look forward to receiving your revised manuscript.

Kind regards,

Andrea Cioffi

Academic Editor

PLOS One

Journal Requirements:

If the reviewer comments include a recommendation to cite specific previously published works, please review and evaluate these publications to determine whether they are relevant and should be cited. There is no requirement to cite these works unless the editor has indicated otherwise.

[Note: HTML markup is below. Please do not edit.]

Reviewers' comments:

Reviewer's Responses to Questions

**Comments to the Author**

Reviewer #3: (No Response)

**********

2. Is the manuscript technically sound, and do the data support the conclusions?

Reviewer #3: Yes

**********

3. Has the statistical analysis been performed appropriately and rigorously?

Reviewer #3: Yes

**********

4. Have the authors made all data underlying the findings in their manuscript fully available?

The PLOS Data policy

Reviewer #3: Yes

**********

5. Is the manuscript presented in an intelligible fashion and written in standard English?

Reviewer #3: Yes

**********

Reviewer #3: Thank you for the opportunity to review the manuscript titled Mapping moral injury: Development and feasibility of the Clinical Interview for Moral Injury—Military Version (CIMI-M) submitted to PLOSOne. I do not believe I was a reviewer on the previous version of the manuscript, so my initial review will be based on the most updated submission.

This reflects an important initiative as moral injury is historically understudied and its investigation outside of military populations is essentially in its infancy. However, there are several notable concerns that I highlight below that I believe will substantively enhance the contribution of this work to the greater psychological literature.

Page 2, first paragraph - this paragraph seems to end more abruptly with a brief description of moral injury-related debates, but this section seems to trail off. I recommend ensuring this section is more cohesive.

'Current moral injury questionnaires, for instance, do not inquire about what moral beliefs were violated, which is relevant for treatment.' This is not true, at least for the Moral Injury Outcome Scale. The first set of items prior to the outcome items reflect the nature of the trauma.

The authors note that a diagnostic clinical interview is not feasible at this stage of construct investigation, which I do not disagree with. However, it is worth examining the following reference to understand and integrate any relevant diagnotic cut-offs that have been proposed (perhaps integration into the Instrument Aim subsection would also be helpful):

Litz, B. T., Walker, H. E., Rusowicz-Orazem, L., Styler, Z. R., Fielstein, E., Darnell, B., ... & Nieuwsma, J. A. (2026). Establishing Clinically Significant Change Benchmarks for the Moral Injury Outcome Scale in VA Behavioral Health Settings. Assessment, 10731911261436687.

Have the authors considered the different populations for which moral injury may be a relevant construct? For example, would there be different presentations and/or interview questions for Veterans versus first responders versus health care workers in general versus civilians, etc? This may be worth describing.

Could the authors describe at the outset (i.e., in the measure development section) how the interview questions differ from those developed for relevant self-report measures of moral injury?

Did the authors consider any moral distress literature, and how were the two constructs differentiated for development of this particular interview?

Example question listed on p. 10 is a closed-ended question, which generally are not as informative in qualitative research. Were most questions like this? Was this considered in their development? Were specific probing questions developed? Why/why not?

There is not a lot of information provided about the theoretical development or background on moral injury; this almost seems tangential to the rest of the paper. I think it would strengthen the manuscript considerably to focus more on the theoretical foundations underlying this work (more than a brief overview).

Qualitative methodology (e.g., how themes were decided upon and extracted) could be more detailed.

'On the DIP, clinical utility was rated M=3.34 (SD=0.47, range 2-4), feasibility M=3.39 (SD=0.55, range 2-4).' Can the authors please define clinical utility, feasibility and acceptability for unfamiliar readers?

When the authors state that Veterans and clinicians were recruited, were the Veterans recruited to take part in the interview and clinicians recruited to deliver the interview to these participants? Please be specific. Were those who conducted the interview adequately prepared and trained in a systematic way? What were participants' gender and ages? Please provide details.

Subsections for findings are very succinct, sometimes only including 1-2 sentences, at the expense of important information. Could some quotes from the table be included in text to improve the flow of the narrative?

Often, the authors mention changes to the CIMI-M without detailing these. For example, 'Revisions included the rewording of some questions, additional follow-up questions, and a new question about self-worth.' Can you please include further information?

'section 4: desired change and readiness for change' - This is not described earlier in the manuscript, but is interesting - explanation for this section is warranted.

It would be extraordinarily helpful for the interview and its instructions to be provided to interested readers to fully understand the manuscript and the development of the interview.

**********

what does this mean?). If published, this will include your full peer review and any attached files.

If you choose “no”, your identity will remain anonymous but your review may still be made public.

**Do you want your identity to be public for this peer review?**  For information about this choice, including consent withdrawal, please see our Privacy Policy

Reviewer #3: No

**********

[NOTE: If reviewer comments were submitted as an attachment file, they will be attached to this email and accessible via the submission site. Please log into your account, locate the manuscript record, and check for the action link "View Attachments". If this link does not appear, there are no attachment files.]

To ensure your figures meet our technical requirements, please review our figure guidelines: https://journals.plos.org/plosone/s/figures

You may also use PLOS’s free figure tool, NAAS, to help you prepare publication quality figures: https://journals.plos.org/plosone/s/figures#loc-tools-for-figure-preparation.

NAAS will assess whether your figures meet our technical requirements by comparing each figure against our figure specifications.

# Author comment: Author response to Decision Letter 2

**DOI:** 10.1371/journal.pone.0359553.r004

24 Aug 2026

Please see the attached document 'response to reviewers'.

Attachment Submitted filename: Response_to_Reviewers_auresp_2.docx

# Peer review: Decision Letter 2

**DOI:** 10.1371/journal.pone.0359553.r005

**Authors:** Andrea Cioffi

16 Sep 2026

Mapping Moral Injury: Development and Feasibility of the Clinical Interview for Moral Injury—Military Version (CIMI-M).

PONE-D-26-06429R2

Dear Dr. de Goede,

We’re pleased to inform you that your manuscript has been judged scientifically suitable for publication and will be formally accepted for publication once it meets all outstanding technical requirements.

Within one week, you’ll receive an e-mail detailing the required amendments. When these have been addressed, you’ll receive a formal acceptance letter and your manuscript will be scheduled for publication.

An invoice will be generated when your article is formally accepted. Please note, if your institution has a publishing partnership with PLOS and your article meets the relevant criteria, all or part of your publication costs will be covered. Please make sure your user information is up-to-date by logging into Editorial Manager at Editorial Manager® and clicking the ‘Update My Information' link at the top of the page. For questions related to billing, please contact billing support.

If your institution or institutions have a press office, please notify them about your upcoming paper to help maximize its impact. If they’ll be preparing press materials, please inform our press team as soon as possible -- no later than 48 hours after receiving the formal acceptance. Your manuscript will remain under strict press embargo until 2 pm Eastern Time on the date of publication. For more information, please contact onepress@plos.org.

Kind regards,

Andrea Cioffi

Academic Editor

PLOS One

Additional Editor Comments (optional):

Reviewers' comments:

Reviewer's Responses to Questions

**Comments to the Author**

Reviewer #3: All comments have been addressed

**********

2. Is the manuscript technically sound, and do the data support the conclusions?

Reviewer #3: Yes

**********

3. Has the statistical analysis been performed appropriately and rigorously?

Reviewer #3: Yes

**********

4. Have the authors made all data underlying the findings in their manuscript fully available?

The PLOS Data policy

Reviewer #3: Yes

**********

5. Is the manuscript presented in an intelligible fashion and written in standard English?

Reviewer #3: Yes

**********

Reviewer #3: (No Response)

**********

what does this mean?). If published, this will include your full peer review and any attached files.

If you choose “no”, your identity will remain anonymous but your review may still be made public.

**Do you want your identity to be public for this peer review?**  For information about this choice, including consent withdrawal, please see our Privacy Policy

Reviewer #3: No

**********

# Editor report: Acceptance letter

**DOI:** 10.1371/journal.pone.0359553.r006

**Authors:** Andrea Cioffi

PONE-D-26-06429R2

PLOS One

Dear Dr. de Goede,

I'm pleased to inform you that your manuscript has been deemed suitable for publication in PLOS One. Congratulations! Your manuscript is now being handed over to our production team.

At this stage, our production department will prepare your paper for publication. This includes ensuring the following:

* All references, tables, and figures are properly cited

* All relevant supporting information is included in the manuscript submission,

* There are no issues that prevent the paper from being properly typeset

You will receive further instructions from the production team, including instructions on how to review your proof when it is ready. Please keep in mind that we are working through a large volume of accepted articles, so please give us a few days to review your paper and let you know the next and final steps.

Lastly, if your institution or institutions have a press office, please let them know about your upcoming paper now to help maximize its impact. If they'll be preparing press materials, please inform our press team within the next 48 hours. Your manuscript will remain under strict press embargo until 2 pm Eastern Time on the date of publication. For more information, please contact onepress@plos.org.

You will receive an invoice from PLOS for your publication fee after your manuscript has reached the completed accept phase. If you receive an email requesting payment before acceptance or for any other service, this may be a phishing scheme. Learn how to identify phishing emails and protect your accounts at https://explore.plos.org/phishing.

If we can help with anything else, please email us at customercare@plos.org.

Thank you for submitting your work to PLOS One and supporting open access.

Kind regards,

PLOS One Editorial Office Staff

on behalf of

Dr. Andrea Cioffi

Academic Editor

PLOS One
